# Prevalence and determinants of emotional, physical, and sexual Intimate Partner Violence in urban Lagos, Nigeria: A cross-sectional study

**DOI:** 10.1371/journal.pone.0359973

**Published:** 2026-10-05

**Authors:** Tope Olubodun, Ajoke Adewole, Victoria Oluwasola Yesufu, Ifeoma P. Okafor, Olorunfemi Akinbode Ogundele, Omobola Yetunde Ojo, Gbemiga Adekunle Ajewole, Mobolanle Rasheedat Balogun

**Affiliations:** 1 Department of Community Medicine and Primary Care, Federal Medical Center Abeokuta, Abeokuta, Ogun State, Nigeria; 2 Department of Primary Care, James Paget University Hospital, Great Yarmouth, Norfolk, United Kingdom; 3 Unified Initiative for a Drug Free Nigeria, Fagba, Lagos State, Nigeria; 4 Department of Community Health and Primary Care, College of Medicine, University of Lagos, Surulere, Lagos State, Nigeria; 5 Department of Community Medicine, University of Medical Sciences, Ondo, Ondo State, Nigeria; Federal Medical Centre Birnin Kudu, NIGERIA

## Abstract

**Background:**

Intimate partner violence (IPV) is a major public health and human rights concern affecting women globally. In Nigeria, entrenched social norms and cultural acceptance of male dominance contribute to its high prevalence. This study assessed the prevalence and determinants of emotional, physical, and sexual IPV among women residing in an urban Local Government Area (LGA) in Lagos, Nigeria.

**Methods:**

This was a cross-sectional study carried out among 502 women who had been married/cohabiting for at least 2 years. Respondents were selected via multi-stage sampling. Data was collected using interviewer-administered questionnaires adapted from the WHO multi-country study questionnaire on women’s health and life experiences. Data was analysed using univariate, bivariate analysis and binary logistic regression.

**Results:**

The mean age of the women was 37.4 ± 8 years. The mean age of women’s partners was 44.4 ± 8.7 years. Overall, 83% of women had ever experienced some form of IPV and 51.8% in the past year. Emotional IPV was most common (78% ever, 47.8% past year), followed by physical (54% ever, 27.5% past year) and sexual IPV (18% ever, 9.4% past year). Cohabiting women and women married for 2–10 years had higher odds of emotional IPV and any IPV. Women with no formal education [aOR 0.15, 95% CI 0.04–0.67] were less likely to experience any IPV. Women whose partners were younger [aOR 6.91, 95% CI 2.21–21.66], engaged in extramarital relationships [aOR 2.30, 95% CI 1.29–4.10], had significantly higher odds of any IPV. Women whose partners drank alcohol daily/nearly every day [aOR 2.18, 95% CI 1.10–4.3] were more likely to be victims of physical IPV. Poor partner communication doubled the likelihood of physical IPV, and women whose partners’ mothers had experienced violence were more likely to experience physical IPV. Women with negative attitudes toward gender roles were at greater risk of experiencing any form of IPV.

**Conclusion:**

This study found a high prevalence of intimate partner violence among women in urban Lagos. Targeted interventions should focus on high-risk groups and be complemented by broader, multi-sectoral efforts to challenge gender norms, empower women, and strengthen legal and health-system responses.

## Introduction

Intimate partner violence (IPV) refers to any behaviour within an intimate relationship that causes physical, psychological or sexual harm to those in the relationship [1]. It includes acts of physical violence such as slapping, hitting, kicking and beating; Sexual violence, including forced sexual intercourse and other forms of sexual coercion; and Emotional (psychological) abuse, such as insults, belittling, and constant humiliation. IPV also includes controlling behaviours, such as isolating a person from family and friends, monitoring their movements, and restricting access to financial resources, employment, education or medical care [1].

IPV is a global public health problem. Globally, over a quarter of women in relationships have experienced physical or sexual violence from an intimate partner [2]. Women in low- and middle-income countries often face a higher risk of IPV; however, this increased vulnerability is shaped by a complex interplay of factors, including entrenched gender norms that legitimise male dominance, unequal power relations within intimate relationships, limited autonomy and decision-making capacity among women, exposure to violence in childhood, harmful alcohol use, and weak legal and institutional protections [3]. Violence against women is embedded within family structures, reinforced by social institutions, and sustained by patriarchal systems that normalise male dominance and female subordination [4].

Though varying from country to country, the burden of IPV in Africa constitutes a significant public health and human rights concern [5]. Ranging from 8.1% in Comoros to 59.9% in Sierra Leone, IPV affects an estimated 46.6% of women in sub-Saharan Africa [5]. The wide range of IPV prevalence rates reported may reflect differences in gender norms, levels of women’s empowerment, legal frameworks, and methodological approaches across settings [6].

Intimate partner violence is a prevalent problem in Nigeria, where data from the 2018 Nigeria Demographic and Health Survey indicate that 36% of women of reproductive age have experienced some form of IPV [7]. A cross-sectional community-based study setting done in Orlu, South-East Nigeria, reported that 56% had experienced some form of IPV [8]. Another study among women attending a primary health centre in Eastern Nigeria reported that over 40% had experienced violence within the last 12 months of the study [9]. Among women attending a health facility in rural Birnin-kudu, northwest Nigeria, 37.0% reported at least one form of violence by their husbands [10]. A study done among women in western Nigeria reported that 73.2% of the respondents had experienced one form of IPV [11]. The varying prevalence reported among studies may reflect different social norms across regions and may also reflect different methodologies in estimating IPV prevalence. Nevertheless, the burden of IPV among Nigerian women is large.

IPV can have grave and devastating effects on women across the life course. These range from physical injuries such as bruises, lacerations, haemorrhage, and concussions to psychological consequences including depression, anxiety, suicidal ideation, and post-traumatic stress disorder [12]. Many women also experience financial hardship, social isolation, and restricted autonomy as a result of abusive relationships [12] In severe cases, IPV may result in death. Additionally, where children are present, they may be directly or indirectly affected through exposure to violence, which can adversely affect their emotional, behavioural, and developmental outcomes [13,14] IPV constitutes a severe violation of women’s human rights; however, it is not inevitable [15]. It can be curtailed through well-designed and effective programmes and policies, guided by relevant data and evidence [15].

Defining the scope and magnitude of IPV is important in planning programmes and designing policies targeted at reducing the burden of IPV. Despite the substantial body of evidence on intimate partner violence in Nigeria, important gaps remain. Many existing studies are facility-based [16–18] or conducted in rural or semi-urban settings [19,20], which may limit generalisability to highly urbanised contexts such as Lagos. Furthermore, while several studies report overall IPV prevalence, fewer have examined the distinct patterns and determinants of emotional, physical, and sexual IPV concurrently within the same population using standardised instruments. In addition, the rapidly changing socio-economic landscape of Lagos, characterised by urbanisation, economic inequality, and shifting gender norms, presents a unique context that may influence both the occurrence and drivers of IPV but remains underexplored in the literature. The selection of variables in this study was guided by the WHO Multi-country Study on Women’s Health and Domestic Violence instrument [21], incorporating established individual, relational, and socio-economic determinants of IPV to enable a comprehensive assessment of IPV, and enhancing comparability with existing national and international research.

This study, therefore, contributes to the existing body of knowledge by providing community-based evidence on the prevalence and determinants of different forms of IPV among women in an urban LGA in Lagos, Nigeria. By disaggregating IPV into its key domains and identifying associated factors, the study offers context-specific insights to inform targeted interventions and policy responses in urban Nigerian settings..

## Methods

### Study area

Lagos State is a state located in the south-western geopolitical zone of Nigeria. Though the smallest in area of Nigeria’s 36 states, Lagos State is regarded as the nation’s economic hub and is the state with the second-highest population [22]. Lagos has a large urban population, which is growing continuously from migration from other states [22]. Lagos 2025 population is estimated at 17,156,400. There are twenty Local Government Areas (LGAs) in Lagos, of which 16 are urban LGAs, and four are rural LGAs.

The study was conducted in Mushin Local Government Area (LGA), which had a projected population of 935,400 in 2022 [23]. The area is characterised by a mix of industrial and commercial activities, with several manufacturing industries located within the LGA. Residents are engaged in diverse occupations, including trading, artisan services, civil service, and private sector employment. There is however a predominance of informal economic activities.

Mushin is also known for its high population density, relatively low socioeconomic status, and elevated crime rates compared to other parts of Lagos State [24,25]. The educational and literacy landscape of Mushin LGA is highly reflective of its low socioeconomic and densely populated urban character. While the area contains an established network of public and private primary and secondary institutions, it faces notable challenges, including high secondary school dropout rates and significant gaps in functional adult literacy [26]. A substantial portion of women in the community possess limited formal schooling, anchoring them primarily to informal petty trading. Mushin LGA provides an important urban context for examining intimate partner violence.

### Study design and study population

This is a community-based cross-sectional study. The study population consists of women who were currently married (customary, religious, or civil) or cohabiting in a non-marital union at the time of data collection, had been in such unions for at least two years, and had resided in Mushin LGA for at least two years. Temporary visitors to the study area were excluded from the study.

### Sample size determination

Sample size was determined using the Cochran sample size formulae for cross-sectional studies, n = Z^2^ pq/ d^2^ [27] where n is the minimum sample size required, Z is the standard normal deviate at 95% confidence interval (1.96), p is the prevalence of IPV in a study in Nigeria (p = 56% [8], q = 1- p, and d is the acceptable error margin (5%). The minimum sample size was calculated as 379 which was increased to 541 after compensating for 30% non-response. At the study-planning stage, this conservative allowance for non-response was made because the sensitive nature of IPV questions and the need to ensure privacy during household interviews were expected to result in refusals, incomplete interviews or inability to conduct some interviews. However, the actual response rate was higher than anticipated.

### Sampling method

A multistage sampling method was used in selecting study participants. In the first stage, three wards were selected from the thirteen wards in Mushin LGA by simple random sampling by balloting. A sampling frame of all the streets in the selected wards was then drawn. In the second stage, a representative number of streets, was selected by simple random sampling by balloting. In the third stage, systematic sampling method was used to select the desired number of houses on each of these streets. In a selected house containing more than one household, all households were identified, and one household was selected by simple random sampling through balloting. In the fourth stage, In the fourth stage, within the selected household, all women who met the eligibility criteria were identified. Where more than one eligible woman was present, one was selected by simple random sampling through balloting.

Using this sampling approach, a total of 554 eligible women were approached, of whom 31 declined participation, and 523 gave informed consent. After excluding 21 questionnaires due to incomplete responses, 502 respondents were included in the final analysis. This gave a response rate of 90.6%.

### Data collection instruments and techniques

Data was collected from 22^nd^ August 2022–5^th^ September 2022. Data was collected using interviewer-administered questionnaires adapted from the WHO multi-country study questionnaire on women’s health and life experiences [21]. The tool is widely validated across diverse settings, uses behaviourally specific items to improve reliability and reduce underreporting, and demonstrates good validity with acceptable internal consistency across IPV domains [21]. The WHO multi-country study questionnaire on women’s health and life experiences was developed and implemented across diverse settings, including low- and middle-income countries in Africa, such as Ethiopia, Namibia and Tanzania [21]. It uses behaviourally specific questions to facilitate disclosure and improve comparability across IPV domains.

Our study questionnaire contained information about the respondent’s sociodemographic background, her reproductive history, her partner’s socio-demographic characteristics and habits, her attitude towards gender roles, her communication with her partner, and her experiences of emotional, physical and sexual IPV and the consequences of IPV.

The study questionnaires were administered by trained female research assistants who had a minimum of an ordinary-level diploma and had experience in quantitative data collection. The data collectors underwent a one-day training conducted by the researchers (TO, AA, and VAY), on principles of quantitative data collection and how to administer the IPV questionnaire, including practice sessions.

Interviews were conducted in English or Yoruba, according to each participant’s preference. Interviews took place in a private area within or around the household, out of the hearing of partners, family members and other persons. Interviews were paused or discontinued if privacy could not be maintained. Prior to data collection, the questionnaire was pretested among 20 eligible women in Isolo LGA, also an urban LGA, The pretest assessed the clarity, appropriateness and administration of the questions, after which necessary modifications were made.

### Outcome variables

The outcome variables included lifetime and past-year experiences of emotional, physical, sexual, and any form of IPV. Specifically, the lifetime outcomes were: ever experienced emotional IPV, ever experienced physical IPV, ever experienced sexual IPV, and ever experienced any IPV. Corresponding past-year outcomes captured experiences of these forms of IPV within the preceding 12 months and were used to examine determinants of IPV. The specific questions used to assess each of these variables are provided in S1 Text.

Each woman was asked seven questions to assess whether she had ever experienced emotional IPV and, for each, whether the experience had occurred in the past 12 months. A “yes” response to any of the lifetime questions classified the woman as having ever experienced emotional IPV, while a “yes” response to any of the past-year questions indicated emotional IPV within the preceding 12 months. The same approach was applied for physical and sexual IPV – seven and three questions were used, respectively, to assess lifetime and past-year experiences.

A woman was classified as having ever experienced any IPV if she reported at least one form of IPV (emotional, physical, or sexual) at any time in her life. Similarly, any IPV in the preceding 12 months referred to reporting at least one of these forms of IPV within the past year.

### Independent variables

The independent variables included sociodemographic characteristics of the women, characteristics of their partners, the women’s attitude toward gender roles, and their communication with their partners. The specific questions used to assess each of these variables are provided in S1 Text.

Sociodemographic characteristics of the woman included the age (in years), marital status, number of years in union, type of marriage, highest level of education, occupation, religion, ethnicity, and average monthly income. The partner’s characteristics include partner’s age (in years), partner’s highest level of education, partner’s occupation, tobacco or smoking history, alcohol drinking history, use of hard drugs, involvement in physical fights, extra-marital relationships, having children outside wedlock, history of the partner’s mother being beaten by her husband, and history of being beaten during childhood.

Women’s attitudes toward gender roles were assessed using ten statements that examined their views on wife-beating and women’s sexual autonomy within marriage. For each statement, responses were recorded as “Yes” (agree) or “No” (disagree), where a “Yes” response indicated agreement with gender-inequitable norms and was scored as 0, while a “No” response reflected rejection of such norms and was scored as 1. The total attitude score for each respondent was summed and converted to a percentage. Scores below 50% were classified as “poor attitude” while scores of 50% or higher were categorised as “good attitude”.

Women’s communication with their partners was assessed using four questions that examined whether couples discussed important aspects of their daily experiences and emotions. Each woman was asked if she and her husband or partner talked about: (1) things that happened to him during the day, (2) his worries or feelings, (3) things that happened to her during the day, and (4) her worries or feelings. Responses were recorded as “Yes” or “No,” with a “Yes” response scored as 1 (indicating positive communication) and a “No” response scored as 0 (indicating absence of communication). The total score for each participant was summed and converted to a percentage. Scores below 50% were classified as “poor communication” while scores of 50% or higher were categorised as “good communication”.

### Data management

All data collected during the study were treated with strict confidentiality and stored securely to protect participants’ privacy. Data were saved on password-protected and encrypted devices, and only authorised members of the research team could access them. Unique codes were used instead of names to ensure anonymity. Access to the dataset was restricted to the principal investigator and designated analysts directly involved in the study.

### Data analysis

Data entry and cleaning were done on Microsoft Excel version 20. Data was imported into and analysed with STATA version 17. Frequency and proportions were generated for categorical variables. Mean and standard deviation were estimated for continuous variables. Bivariate analysis was conducted using the Chi-square test to examine associations between women’s characteristics, partners’ characteristics, attitudes toward gender roles, and couple communication, with experiences of emotional, physical, sexual, and any form of IPV in the preceding 12 months. Results from bivariate analysis are reported in S1 Table. Variables that were statistically significant at *p* ≤ 0.05 in the bivariate analysis were included in a binary logistic regression model to identify determinants of emotional, physical, sexual, and any IPV experienced in the preceding 12 months. The level of significance was set at 5%, and confidence level at 95%.

### Ethics approval and consent to participate

Ethical clearance for this study was obtained from the Lagos University Teaching Hospital, Health Research Ethics Committee. (Approval Number: ADM/DSCST/HREC/APP/4673). Written informed consent was obtained from each respondent, and respondents were not coerced. Questionnaires were coded and did not contain the names of women. Respondents were assured of confidentiality, and the interviews were conducted respectfully and with privacy. Relevant community leaders and representatives were engaged before the study, supported community entry during data collection, and were informed of the study findings after its completion. All procedures were conducted in line with the Principles of Helsinki.

## Results

The mean age of the respondents was 37.4 ± 8 years. Most of the respondents were legally married (60.2%), were in monogamous marriages (76.1%), and had attained secondary school level of education (64.3%). The majority were unskilled workers (44.4%) or semiskilled (49%). Most of the women (62.2%) earned between N20,000 – N39,999 monthly, and about half had between 2–3 children (52.2%). About half of the women were Muslims (50.6%), and half wereChristians (49.4%). The Yoruba ethnicity was the predominant ethnicity (84.9%). (Table 1)

**Table 1:** Social demographic characteristics of respondents (n = 502).

Variable	Frequency (n = 502)	Percentage
**Age (in years)**		
20 - 29	90	17.9
30 - 39	221	44.0
≥ 40	191	38.0
**Mean**	37.4 ± 8.0	
**Marital Status**		
Legally Married	302	60.2
Co-habiting	200	39.8
**Number of years in union**		
2–10	265	52.8
11 - 20	152	30.3
>20	85	16.9
**Mean**	12.4 ± 8.3	
**Type of marriage**		
Monogamous	382	76.1
Polygamous	120	23.9
**Highest level of education**		
No formal education	16	3.2
Primary	84	16.7
Secondary	323	64.3
Post secondary education	79	15.7
**Occupation**		
Unemployed	18	3.6
Unskilled/Manual	223	44.4
Semi-skilled	248	49.4
Skilled	13	2.6
**Religion**		
Christianity	248	49.4
Islam	254	50.6
**Ethnicity**		
Yoruba	426	84.9
Hausa	8	1.6
Ibo	44	8.8
Others	24	4.8
**Average monthly income**		
<N20,000 (<$48.19)	94	18.7
N20,000 – N39,999 ($48.19-$96.38)	302	60.2
>N40,000 (≥$96.39)	106	21.1
**Number of children**		
1	91	18.1
2 −3	262	52.2
≥ 4	149	29.7

$1 USD = ₦415.

Most of the respondents’ partners were aged 35–45 years (47.0%) and >45 years (41.0%). Sixty-two per cent had at least a secondary school level of education, and 84.9% were semi-skilled. Fifteen per cent of the women’s partners smoke/use tobacco, 27.7% take alcohol everyday/nearly every day, and 8.4% use hard drugs. Twenty per cent of the partners have ever had a physical fight with another man, 34.7% had ever had an extra-marital relationship, and 16.1% had children outside marriage/wedlock (83.9%). For more than half of the women, their partner was beaten regularly by his parents/family members while growing up (58.6%). (Table 2)

**Table 2:** Attributes of respondent’s partner (n = 502).

Variable	Frequency (n = 502)	Percentage
**Partner’s age (in years)**		
< 35	60	12.0
35-45	236	47.0
>45	206	41.0
**Mean±SD**	44.4 ± 8.7	
**Partner’s highest level of education**		
No formal education	10	2.0
Primary	73	14.5
Secondary	315	62.7
Higher	104	20.7
**Partner’s occupation**		
Unemployed	4	0.8
Unskilled	41	8.2
Semi-skilled	426	84.9
Skilled	31	6.2
**Tobacco or smoking history of Partner**		
Smokes/use tobacco	76	15.1
Does not smoke/use tobacco	426	84.9
**Alcohol drinking history of Partner**		
Every day or nearly everyday	139	27.7
Once or twice a week	118	23.5
Never/Less than once a week	245	48.8
**Partner uses of hard drugs**		
Uses hard drugs	42	8.4
Does not use hard drugs	460	91.6
**Partner involved in physical fight with another man**		
No	400	79.7
Yes	102	20.3
**Partner having extra-marital relationship**		
No	261	52
Yes	174	34.7
Don’t know	67	13.3
**Partner had children outside wedlock during marriage**		
No	421	83.9
Yes	81	16.1
**History of partner’s mother being beaten by her husband**		
No	282	56.2
Yes	20	4.0
Don’t know	200	39.8
**Partner was beaten regularly by parents or members of his family while growing up**		
No	188	37.5
Yes	294	58.6
Don’t know	20	4.0

Most women had positive attitudes towards gender roles (98.6%) (Table 3) and most had good communication with their partners (84.1%) (Table 4). These variables were subsequently examined as potential determinants of IPV.

**Table 3:** Attitudes towards gender roles among respondents (n = 502).

Response	Frequency (n = 502)	Percentage
A man has a good reason to hit his wife if she does not complete her household work to his satisfaction		
No	498	99.2
Yes	4	0.8
A man has a good reason to hit his wife if she disobeys him		
No	491	97.8
Yes	11	2.2
A man has a good reason to hit his wife if she refuses to have sexual relations with him		
No	493	98.2
Yes	9	1.8
A man has a good reason to hit his wife if she asks him whether he has other girlfriends		
No	493	98.2
Yes	9	1.8
A man has a good reason to hit his wife if he suspects that his wife is unfaithful to him		
No	457	91.0
Yes	45	9.0
A man has a good reason to hit his wife if he finds out that his wife is unfaithful to him		
No	262	52.2
Yes	240	47.8
A married woman can refuse to have sex with her husband if she does not want to		
No	159	31.7
Yes	343	68.3
A married woman may refuse to have sex with her husband if he is drunk		
No	18	3.6
Yes	484	96.4
A married woman can refuse to have sex with her husband if she is sick		
No	111	22.1
Yes	391	77.9
A married woman can refuse to have sex with her husband if he mistreats her		
No	497	99.0
Yes	5	1.0
**Attitude towards gender roles**		
**Negative attitude**	**7**	**1.4**
**Positive attitude**	**495**	**98.6**

**Table 4:** Communication between respondent and her partner (n = 502).

Husband/partner discuss the following topics together	Frequency (n = 502)	Percentage
**Things that have happened to him in the day**		
No	77	15.3
Yes	425	84.7
**His worries or feelings**		
No	76	15.1
Yes	426	84.9
**Things that happen to you during the day**		
No	50	10
Yes	452	90
**Your worries or feelings**		
No	50	10
Yes	452	90
**Overall communication with partner**		
**Poor communication**	80	15.9
**Good communication**	422	84.1

Among the emotional IPV items, being insulted or made to feel bad was the most frequently reported experience, both during the women’s lifetime (69.9%) and in the preceding 12 months (36.1%). This was followed by being made to feel that they were not good enough (32.9% lifetime and 19.1% in the preceding 12 months) and being deliberately scared or intimidated (35.1% and 17.9%, respectively).

For physical IPV, being slapped or having an object thrown at them was the most commonly reported lifetime experience (40.6%), followed by being hit with a fist (32.7%) and being pushed or shoved (25.1%). In the preceding 12 months, 16.5% reported being slapped or having an object thrown at them, while 16.3% had been hit with a fist.

Regarding sexual IPV, 16.7% of women had ever been physically forced to have sexual intercourse, 8.4% had intercourse because they were afraid of what their partner might do, and 2.6% had been forced to perform a degrading or humiliating sexual act. The corresponding prevalence estimates in the preceding 12 months were 8.0%, 4.2% and 1.8%, respectively (Table 5).

**Table 5:** Prevalence of specific acts of emotional, physical, and sexual intimate partner violence (n = 502).

Specific Acts of Violence	Frequency	Percentage (%)
**Lifetime experience of Emotional IPV**		
Partner insulted her or called her names	351	69.9
Partner embarrassed her in public	159	31.7
Partner did things to make her afraid	176	35.1
Partner threatened to hurt her	80	15.9
She feels nothing does is good enough for her partner	165	32.9
Partner sent her packing out of the house	49	9.8
Partner walked out of the house for at least a day	90	17.9
**Past 12 months’ experience of Emotional IPV**		
Partner insulted her or called her names	181	36.1
Partner embarrassed her in public	86	17.1
Partner things to make her afraid	90	17.9
Partner threatened to hurt her	41	8.2
She feels nothing she does is good enough for her partner	96	19.1
Partner sent her packing out of the house	16	3.2
Partner walked out of the house for at least a day	55	11
**Lifetime experience of Physical IPV**		
Partner slapped her	204	40.6
Partner pushed or shoved her	126	25.1
Partner hit her with an open hand or fist	164	32.7
Partner hit her with something else that could hurt her	54	10.8
Partner beat her up	119	23.7
Partner choked her	10	2
Partner threatened to use or actually used a knife or other weapon against her	9	1.8
**Past 12 months experience of Physical IPV**		
Partner slapped her	83	16.5
Partner pushed or shoved her	58	11.6
Partner hit her with an open hand or fist	82	16.3
Partner hit her with something else that could hurt her	31	6.2
Partner beat her up	56	11.2
Partner choked her	6	1.2
Partner threatened to use or actually used a knife or other weapon against her	7	1.4
**Lifetime Experience of Sexual IPV**		
Partner forced her to have sexual intercourse when she did not want to	84	16.7
She had sexual intercourse with her partner because she was afraid	42	8.4
Partner forced her to perform a sexual act that she found degrading	13	2.6
**Past 12 months’ experience of Sexual IPV**		
Partner forced her to have sexual intercourse when she did not want to	40	8
She had sexual intercourse with her partner because she was afraid	21	4.2
Partner forced her to perform a sexual act that she found degrading	9	1.8

About eight in ten women (78.3%) had ever experienced emotional IPV, and about five in ten women (47.8%) had experienced this in the preceding 12 months. Fifty-four per cent of women had ever experienced physical IPV, and 27.50% experienced physical IPV in the preceding 12 months. Eighteen per cent of women had ever experienced sexual IPV, and 9.4% experienced sexual IPV in the preceding 12 months. Eighty-three per cent of women had ever experienced any IPV, and 51.8% experienced any IPV in the preceding 12 months (Fig 1).

**Fig 1:**
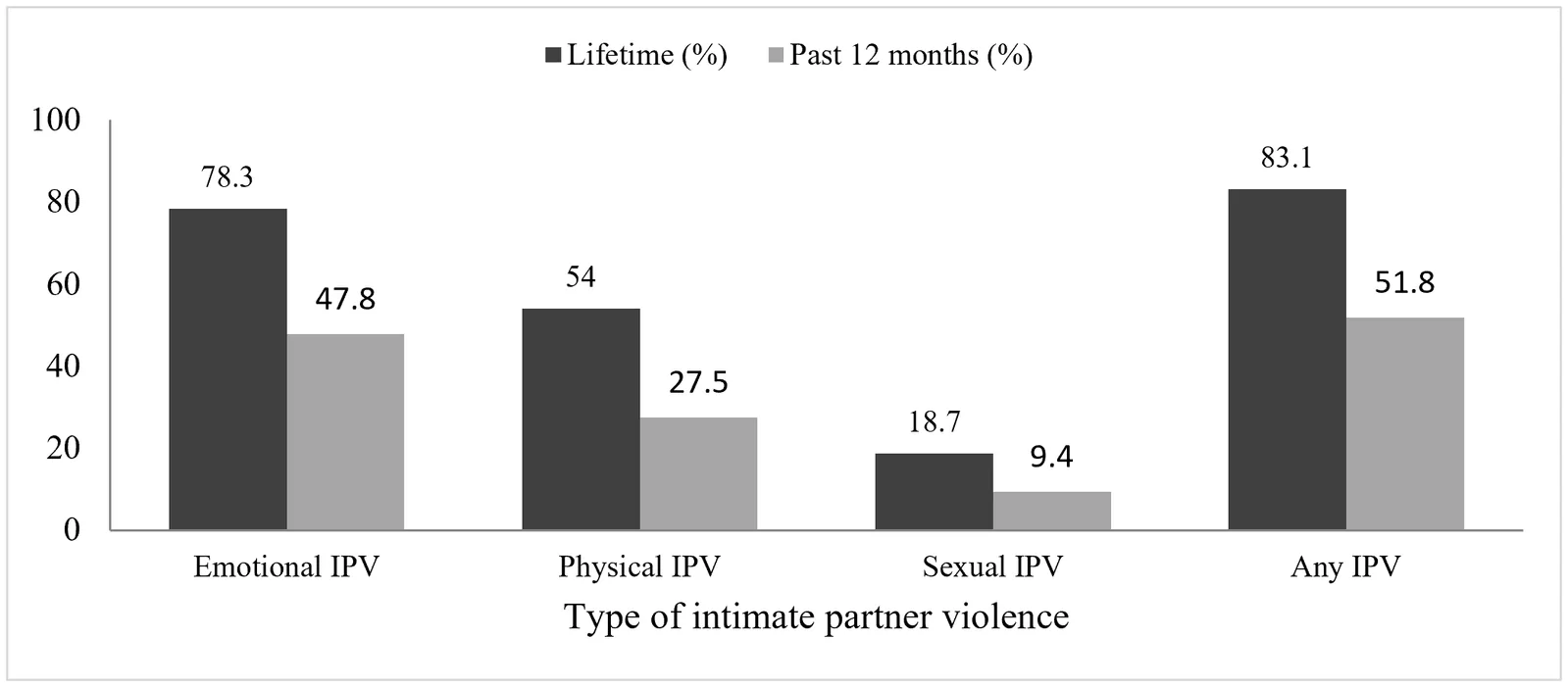
Lifetime and 12-month prevalence of intimate partner violence among respondents (n = 502).

Women aged 20–29 years were less likely to have experienced emotional IPV [aOR 0.30, 95% CI 0.10–0.89] than women ≥ 45 years old. Women that were cohabiting [aOR1.80, 95% CI 1.12–2.90], women married for two to 10 years [aOR 2.49, 95% CI 1.02–6.10], women of Yoruba ethnicity [aOR 2.48, 95% CI 1.30–4.71], women whose partner was ≤ 45 years old [aOR 5.79, 95% CI 1.95–17.22] and women whose partners had extra-marital relationships [aOR 1.86, 95% CI 1.02–3.39] were more likely to experience emotional IPV (Table 6).

**Table 6:** Determinants of emotional IPV, physical IPV, sexual IPV, and any IPV (in last 12 months).

	Emotional IPV aOR(95% CI)	Physical IPV aOR(95% CI)	Sexual IPV aOR(95% CI)	Any IPV aOR(95% CI)
**Age in years**				
20-29	0.30(0.10-0.89)*	1.39(0.51-3.76)	0.42(0.07-2.60)	0.29(0.09-0.88)*
30-39	0.55(0.26-1.17)	1.68(0.78-3.62)	1.441(0.39-5.38)	0.51(0.24-1.10)
≥40	1	1	1	1
**Marital status**				
Legally Married	1	1	1	1
Co-habiting	1.80(1.12-2.90)*	2.20(1.27-3.83)*	0.80(0.36-1.79)	2.33(1.41-3.87)*
**Number of years spent in marriage**				
2 - 10 years	2.49(1.02-6.10)*	1.03(0.31-3.38)	2.903(0.27-30.83)	1.55(0.62-3.84)
11 - 20 years	1.64(0.79-3.41)	1.01(0.36-2.80)	3.12(0.35-28.08)	1.27(0.62-2.62)
>20 years	1	1	1	1
**Type of marriage**				
Monogamous			1	
Polygamous			0.41(0.16-1.09)	
**Education**				
No formal education		0.46(0.06-3.59)		0.15(0.04-0.67)*
Primary education		1.10(0.37-3.31)		0.52(0.23-1.20)
Secondary education		0.7(0.3-1.63)		0.57(0.31-1.06)
Post secondary		1		1
**Religion**				
Christianity		1.61(0.96-2.68)		
Islam		1		
**Ethnicity**				
Yoruba	2.48(1.30-4.71)*	1.07(0.49-2.34)	4.39(0.93-20.70)	2.22(1.18-4.20)*
Non-Yoruba	1	1	1	1
**Occupation**				
Unemployed	0.72(0.14-3.74)			
Unskilled	0.93(0.25-3.50)			
Semi-skilled	0.73(0.20-2.68)			
Skilled	1			
**Average monthly income**				
<N20,000 (<$48.19)	1.01(0.47-2.18)	2.09(0.93-4.70)	0.80(0.21-3.00)	1.36(0.67-2.77)
N20,000 – N39,999 ($48.19-$96.38)	1.20(0.68-2.13)	1.52(0.77-3.02)	1.493(0.56-4.07)	1.31(0.75-2.30)
>N40,000 (≥$96.39)	1	1	1	1
**Number of children**				
1	1.88(0.80-4.45)	3.69(1.29-10.59)*	1.43(0.36-5.64)	2.37(0.96-5.86)
2-3	1.37(0.73-2.55)	2.12(0.92-4.88)	0.99(0.30-2.91)	1.18(0.62-2.23)
>3	1	1	1	1
**Partner’s age (in years)**				
<35	5.79(1.95-17.22)*		9.20(1.72-49.18)*	6.91(2.21-21.66)*
35-45	3.70(1.81-7.56)*		2.06(0.61-6.89)	4.72(2.28-9.80)*
>45	1		1	1
**Partner’s highest level of education**				
No formal education		0.48(0.05-4.64)		
Primary education		0.50(0.16-1.56)		
Secondary education		1.38(0.63-3.05)		
Post secondary education		1		
**Partner’s occupation**				
Unemployed/Unskilled			0.31(0.06-1.51)	
Semi-skilled/Skilled			1	
**Tobacco or smoking history of partner**				
Smokes/use tobacco	1.18(0.56-2.51)	0.84(0.38-1.88)	1.36(0.49-3.77)	1.01(0.49-2.45)
Does not smoke/use tobacco	1	1	1	1
**Alcohol drinking history of partner**				
Every day or nearly every day	1.76(0.98-3.14)	2.18(1.10-4.3)*	1.33(0.50-3.57)	1.39(0.75-2.55)
Once or twice per week	1.364(0.81-2.3)	1.71(0.91-3.23)	1.37(0.58-3.25)	1.49(0.84-2.47)
Never/less than a week	1	1	1	1
**History of partner using hard drugs**				
Uses hard drugs	1.09(0.39-3.03)	1.51(0.57-3.97)	1.15(0.37-3.64)	0.99(0.32-3.02)
Does not use hard drugs	1	1	1	1
**Partner involved in physical fight with another man**				
No	1	1	1	1
Yes	1.49(0.79-2.60)	2.57(1.39-4.72)*	1.45(0.61-3.41)	2.62(1.37-5.00)*
**Partner has extra-marital relationship**				
No	1	1	1	1
Yes	1.86(1.02-3.39)*	2.30(1.29-4.10)*	3.00(1.37-6.57)*	2.44(1.29-4.62)*
Don’t know	1.46(0.76-2.80)	1.27(0.58-2.75)	0.59(0.15-2.29)	1.55(0.80-3.02)
**Partner had children outside wedlock/marriage**				
No	1			1
Yes	1.68(0.79-3.57)			1.41(0.64-3.11)
**Communication with partner**				
Poor communication	1.76(0.94-3.31)	2.37(1.26-4.46)*	0.95(0.39-2.29)	1.61(0.83-3.13)
Good communication	1	1	1	1
**History of partners mother being beaten by her husband**				
No	1	1		1
Yes	0.85(0.30-2.41)	5.98(1.91-18.67)*		1.73(0.58-5.19)
Don’t know	1.04(0.66-1.63)	1.65(0.98-2.79)		1.06(0.66-1.70)
**Attitude towards gender roles**				
Negative attitude			8.27(0.99-69.20)	11.87(1.13-125.04)*
Positive attitude			1	1

*p < 0.05.

Women who were cohabiting [aOR 2.20, 95% CI 1.27–3.83], women who had only one child [aOR 3.69, 95% CI 1.29–10.59], women whose partner’s drinking alcohol every day [aOR 2.18, 95% CI 1.10–4.3], women whose partner had been involved in a physical fight with another man [aOR 2.57, 95% CI 1.39–4.72], women whose partners had extra-marital relationships [aOR 2.30, 95% CI 1.29–4.10], women with poor communication with their partners [aOR 2.37, 95% CI 1.26–4.46)], as well as women with history of partners mother being beaten [aOR 5.98, 95% CI 1.91–18.67] were more likely to experience physical IPV (Table 6).

Women whose partners were <35 years old (aOR 9.20, 95% CI 1.72–49.18) and women whose partners had extra-marital relationships (aOR 3.00, 95% CI 1.37–6.57) were more likely to experience sexual IPV compared with women whose partners were >45 years old and whose partners did not have extra-marital relationships (Table 6).

Women aged 20−29 years were less likely to have experienced any IPV [aOR 0.29, 95% CI 0.09–0.88] than women 45 years or older. Women that were cohabiting [aOR 2.33, 95% CI 1.41–3.87], women of the Yoruba tribe [aOR 2.22, 95% CI 1.18–4.20], women whose partners were < 35 years of age [aOR 6.91, 95% CI 2.21–21.66] women whose partner had been involved in a physical fight with another man [aOR 2.62, 95% CI 1.37–5.00], women whose partners had extra-marital relationships [aOR 2.44, 95% CI 1.29–4.62], and women with negative attitude towards gender roles [aOR 11.87, 95% CI 1.13–125.04] were more likely to experience any IPV. Women with no formal education [aOR 0.15, 95% CI 0.04–0.67] were less likely to experience any IPV than women with post-secondary education (Table 6).

## Discussion

This study aimed to assess the prevalence and determinants of emotional, physical, and sexual intimate partner violence among women in an urban Local Government Area in Lagos, Nigeria. The findings revealed a high prevalence of IPV in this setting. Emotional IPV was the most common form, with nearly eight in ten women reporting lifetime experience and about half within the preceding year. More than half had ever experienced physical IPV, with about one in four reporting it in the past year, while approximately one in five had experienced sexual IPV, and about one in ten had experienced it within the preceding year. Overall, the majority of women had experienced at least one form of IPV in their lifetime, and about half had experienced IPV within the past year.

Cohabiting women, and women married for 2–10 years had higher odds of emotional and any IPV. Women with no formal education were less likely to experience any IPV. Women with only one child were more likely to experience physical IPV than women with four or more children. Partner-related factors strongly influenced IPV risk: women whose partners were younger, drank alcohol daily, or engaged in extramarital relationships had significantly higher odds of IPV. Poor partner communication doubled the likelihood of physical IPV, and women whose partners’ mothers had experienced violence were six times more likely to experience physical IPV. Additionally, women with negative attitudes toward gender roles were at greater risk of experiencing any form of IPV.

The prevalence of IPV was high in this study as majority of the women had experienced any IPV at least once in their lifetime, and half in the preceding year. In contrast, a systematic review from Malaysia reported lower prevalence rates, ranging from 5% to 35% across included studies [28]. Similarly, findings from the Ethiopian Demographic and Health Survey indicated an overall prevalence of approximately 30% [29]. The differences in findings, from our study, may reflect variation in cultural norms, gender relations, social acceptance of violence, and reporting practices, across settings. The Nigeria Demographic Health Survey 2018 put the lifetime prevalence of IPV among ever-partnered women aged 15–49 years at 36% [7]. This prevalence reported in our study is much higher than this. This disparity may partly reflect the local context of the study area – an urban community in Lagos State characterised by high population density, socioeconomic challenges, and social complexity – which may influence the occurrence and reporting of IPV. However, IPV is a multifactorial phenomenon driven by a complex interplay of individual, relational, community, and structural factors, including gender norms, power imbalances, and societal acceptance of violence. Given the far-reaching physical, psychological, and social consequences of IPV on women, families, and communities, urgent and coordinated action is needed to address this pervasive public health and human rights issue.

Emotional IPV was the most prevalent form of IPV in this study, followed by physical and sexual IPV. This pattern mirrors findings from sub-Saharan Africa and other low- and middle-income countries, where emotional abuse often exceeds physical or sexual violence. For example, in a study in Pakistan, the self-reported past-year prevalence of psychological abuse was 83.6%, for physical violence was 56.3, and for sexual violence was 53.4% [30] Similarly, a systematic review and meta-analysis of studies in sub-Saharan Africa showed that emotional violence was the most prevalent form of IPV [31]. Emotional IPV may be more pervasive because it is less recognised as abuse and often normalised within patriarchal contexts. Despite its invisibility, it has serious psychological and physical health consequences, underscoring the need for IPV prevention programs to address emotional as well as physical and sexual forms.

In our study, women who had been in union for 2–10 years were more likely to experience IPV than those in union for over 20 years. This finding may reflect a combination of factors beyond relationship duration alone. Early years of union are often characterised by relationship instability, economic pressures, and ongoing negotiation of roles and power dynamics, which may increase vulnerability to conflict and violence. However, this association may also be influenced by age and cohort effects, as women in shorter unions are more likely to be younger and may differ in their awareness, perception, and willingness to report different forms of IPV. Increased public awareness and evolving social norms around IPV may also contribute to greater recognition and disclosure among younger women compared to older cohorts. While some studies have reported higher IPV prevalence in shorter relationships [32], this relationship should be interpreted cautiously, as it is likely shaped by interacting individual, relational, and contextual factors. Similar patterns have been observed in studies examining severity of violence, including weapon use, within specific durations of union [33].

Regarding type of union, women who were cohabiting were more likely to experience emotional IPV, Physical IPV and any IPV. The relative instability and lack of social/legal protection often associated with informal unions, as well as the weaker commitment and relationship cohesion, may explain our findings. Women that are cohabiting may also have limited access to family or community support structures, which can increase their susceptibility to partner violence. In a study based on the Demographic Health Survey in eight countries in sub-Saharan Africa, women who were co-habiting were more likely to experience IPV [34]. Also, in a study in South Africa, all types of IPV were higher among women that were cohabiting [35].

Women with only one child were more likely to experience IPV in this study. This association may reflect a combination of factors. Women with fewer children are more likely to be in earlier stages of their relationships, where instability, adjustment challenges, and negotiation of roles may increase vulnerability to conflict and violence. The transition to parenthood, particularly with the first child, can also introduce significant emotional, financial, and caregiving stress, which may heighten the risk of IPV. In addition, in some settings, women’s perceived social standing within marriage may be influenced by childbearing, with those having more children sometimes viewed as having greater stability within the union. While reproductive factors have been associated with IPV in previous studies [36,37], these findings should be interpreted cautiously, as the relationship between parity and IPV is complex and context-dependent.

Women whose partners drank alcohol every day/nearly every day were twice as likely to experience IPV. This finding has been reported in other studies [16] A meta-analysis showed that heavy or frequent alcohol consumption by male partners increased the risk of IPV [38] In a study among pregnant women in Nigeria, partner alcohol use increased the likelihood of experience of IPV by twofold [16] Alcohol use may lower inhibitions, impair judgment, and increase aggression, escalating conflicts within relationships. It is also often associated with financial stress and unemployment, which can further heighten the risk of partner violence. Our study, however, did not show any associations between smoking, hard drugs use and IPV, unlike other published studies [39,40] This may be because the women whose partners smoked or used hard drugs were few in our study and the sample size may have been underpowered to detect a statistically significant association.

Women whose partners had extramarital relationship were more likely to experience all forms of IPV. This finding aligns with evidence from previous studies showing that partner infidelity or suspicion of infidelity is a strong predictor of IPV [41,42]. Extramarital affairs often generate jealousy, mistrust, and conflict within relationships, which may trigger emotional or physical violence. Women whose partners mothers experienced IPV were six times more likely to experience physical IPV. This finding is supported by a previous study in India [43] and also in the Philippines [44]. This highlights how witnessing or normalizing violence within the family can reinforce harmful gender norms and perpetuate the cycle of IPV across generations.

Women that had poor communication with their partners were twice as likely to experience physical IPV than their counterparts. Effective partner communication fosters understanding and mutual respect, while poor communication can breed conflict and emotional distance, increasing the risk of physical violence. Promoting couple communication and conflict-resolution skills through counseling and community-based interventions may help reduce IPV and strengthen relationship quality. Women with negative attitude towards gender roles were more likely to be victims of any IPV. Similarly, an analysis of the NDHS showed that women that justified wife beating were more likely to be victims of IPV [45].

In our study, women with no formal education were less likely to experience IPV. This finding contrasts with evidence from East Africa [46] and India [47] [48], where women lacking education were more likely to be victims of IPV. However, it aligns with a study from Ethiopia that reported lower odds of IPV among women whose educational level was lower than their partners’ [48]. A plausible explanation for our finding is that women with limited education may adopt more traditional or submissive gender roles, feeling less empowered to challenge or question their husbands’ decisions. Such deference might reduce overt conflict or disagreement within relationships, thereby lowering the likelihood of IPV.

### Strengths and limitations

This study provides valuable evidence on the prevalence and determinants of IPV among women in an urban Nigerian setting, where population-based data remain scarce. Data were collected using standardized and validated tools consistent with the World Health Organization’s IPV research guidelines, ensuring comparability with other studies. The inclusion of a broad range of socio-demographic and relationship variables allowed for a comprehensive assessment of determinants. Exploring the physical, emotional, and sexual dimensions of IPV provided a nuanced understanding of its multifaceted impact on women’s wellbeing.

This study however has some limitations. As a cross-sectional study, causal relationships cannot be established. Self-reported data may be subject to recall and social desirability biases, particularly given the sensitive nature of IPV. To minimise these biases, trained female research assistants conducted the interviews privately, assured participants of confidentiality and used behaviourally specific questions adapted from the WHO multi-country questionnaire. Questions were also structured around defined reference periods, including lifetime and the preceding 12 months, to facilitate recall. Finally, the study was conducted in one urban Local Government Area of Lagos, which may limit the generalizability of findings to rural or other socio-cultural settings in Nigeria. Nonetheless, the findings make an important contribution to understanding the burden, patterns, and correlates of IPV among urban Nigerian women and can inform targeted interventions and policy responses.

### Implications for policy and practice

The high prevalence of IPV observed in this study underscores the need for comprehensive, multi-level interventions that address the individual, relational, and structural drivers of violence. Findings indicate that IPV in this setting is strongly influenced by relationship dynamics and partner behaviours, highlighting the importance of interventions that extend beyond women-focused approaches. At the health-system level, integrating routine IPV screening into reproductive, maternal, and primary healthcare services is essential for early identification and referral. Given the high burden of emotional IPV, healthcare providers should be trained to recognise non-physical forms of abuse and provide appropriate psychosocial support.

The strong association between IPV and partner-related factors—such as alcohol use, involvement in physical fights, and extramarital relationships—points to the need for targeted male engagement strategies, including programmes that address harmful alcohol use, promote positive masculinity, and challenge norms supporting male dominance and aggression. In addition, the increased risk of IPV among cohabiting women, those in early-stage unions, and those reporting poor partner communication underscores the need for relationship-level interventions that strengthen communication, conflict resolution, and negotiation skills. Addressing the intergenerational transmission of violence through early-life and family-based interventions is also critical.

At the societal level, sustained community engagement is needed to transform harmful gender norms and improve awareness of women’s rights and available support services, alongside strengthening the enforcement of legal protections against domestic violence. Overall, interventions should adopt a holistic approach that addresses the broader social and relational context of IPV, and should not only focus on isolated risk factors, in order to achieve meaningful and sustainable reductions in IPV in urban Nigerian settings.

## Conclusion

This study revealed a high prevalence of IPV among women residing in an urban area of Lagos, Nigeria, with emotional violence being the most common form, followed by physical and sexual IPV. Multiple individual, relational, and contextual factors were significantly associated with IPV. These findings highlight that IPV in urban Nigerian settings is driven not only by personal behaviors but also by deep-rooted social and cultural norms that perpetuate gender inequality and normalize violence within relationships. Addressing IPV therefore requires comprehensive, multi-sectoral interventions that combine community education, empowerment of women, promotion of gender-equitable norms, and strengthening of legal and health-system responses. Sustained policy commitment and evidence-based programming are essential to reduce the burden of IPV and safeguard the health and wellbeing of women and families.

## Supporting information

S1 Text Study questionnaire. (PDF)

S1 Table Bivariate analysis of factors associated with IPV. (DOCX)
