# Supplementary material for: Prevalence and determinants of emotional, physical, and sexual Intimate Partner Violence in urban Lagos, Nigeria: A cross-sectional study
Source: PLoS One. 2026 Oct 5;21(10):e0359973. doi: 10.1371/journal.pone.0359973 (PMC13638058; doi:10.1371/journal.pone.0359973)
Supplement: Supplementary file 1 [file pone.0359973.s001.pdf]

WOMENS HEALTH AND LIFE EVENTS 3

Interviewer's Name

____

1. Date of interview

yyyy-mm-dd

2 Are you willing to participate in this survey

- ◯ OK

3 Local Government Area

- ◯ Mushin/ Idi Oro
- ◯ Mushin/ Kayode Fadeyi
- ◯ Mushin/Omodigbo
- ◯ Mushin Babalosha
- ◯ Badagry 1
- ◯ Badagry 2
- ◯ Badagry 3
- ◯ Badagry 4

SECTION 1: SOCIODEMOGRAPHY OF RESPONDENT

____

1 How old were you on your last birthday?

Age in Years

____

2 Marital Status

[

- ◯ LEGALLY MARRIED......2
- ◯ CO-HABITING.....3

3 Type of Marriage/Cohabiting

- ◯ MONOGAMOUS....... 1
- ◯ POLYGAMOUS....... 2

3B Number of Years in marriage

ANY WOMAN WHO HAS NOT BEEN MARRIED THAN A YEAR SHOULD BE EXCLUDED FROM THIS STUDY

____

4 How Long have you been living continuously in this community

(ANSWERS SHOULD BE IN YEARS)

____

5 What is the highest level of education that you have achieved?

- ◯ NO FORMAL EDUCATION.....1
- ◯ PRIMARY....2
- ◯ SECONDARY....3
- ◯ OTHER POST SECONDARY eg. OND 4
- ◯ HND/UNIVERSITY 5
- ◯ MASTERS/PHD..... 6

6 Occupation

____

7 Occupation

- ◯ UNEMPLOYED....... 1
- ◯ STUDENT 2
- ◯ UNSKILLED/MANUAL e.g cleaner, petty trader...3
- ◯ SEMI-SKILLED e.g. hairdresser, tailor, large trader.....4
- ◯ SKILLED e.g. banker, doctor... 5

8 Religion

- ◯ CHRISTIANITY.... 1
- ◯ ISLAM. .. 2
- ◯ TRADITIONAL... 3
- ◯ OTHERS......4

9 Ethnic group

- YORUBA...... 1
- HAUSA. 2
- IBO..... 3
- EGUN.....4
- OTHERS.....5

9B If Others please specify

10 How much is your average monthly income

SECTION 2: RESPONDENT AND HER COMMUNITY

11 In the past 4 weeks, has someone from this household been the victim of a crime in this neighbourhood, such as a robbery or assault?

- YES........1
- NO 2

12 In the past 6 months, has someone from this household been the victim of a crime in this neighbourhood, such as a robbery or assault?

- YES......1
- NO 2

13 Do neighbours in this community generally tend to know each other well?

- YES. 1
- NO ..2

14 Do any of your family of birth live close enough that you can easily see/visit them?

- YES.....1
- NO 2

15 How often do you see or talk to a member of your family of birth?

- AT LEAST ONCE A WEEK.... 1
- AT LEAST ONCE A MONTH. ...2
- AT LEAST ONCE A YEAR....3
- NEVER/(HARDLY EVER).... 4

16 When you need help or have a problem, can you usually count on members of your family of birth for support?

- ◯ YES.... 1
- ◯ NO 2

SECTION 3: REPRODUCTIVE HISTORY

---

18 How many children do you have?

____

SECTION 4: CURRENT OR MOST RECENT PARTNER SECTION 4: CURRENT OR MOST RECENT PARTNER

---

19 I would now like you to tell me a little about your current/ most recent partner. How old was your husband/partner on his last birthday?

____

20 What is the highest level of education that he achieved?

- ◯ NO FORMAL EDUCATION.... 1
- ◯ PRIMARY 2
- ◯ SECONDARY ... 3
- ◯ HIGHER.... 4
- ◯ DON'T KNOW 9

21 What kind of work does your husband/partner normally do?

---

22 What kind of work does/did he normally do?

- ◯ UNEMPLOYED.... 1
- ◯ STUDENT 2
- ◯ UNSKILLED/MANUAL e.g. cleaner, labourer. 3
- ◯ SEMI-SKILLED e.g. tailor, mechanic, barber, driver. 4
- ◯ SKILLED e.g banker.....5

23 How often does your partner smoke cigarettes or use any form of tobacco?

- EVERY DAY OR NEARLY EVERY DAY ... 1
- ONCE OR TWICE A WEEK... 2
- 1-3 TIMES IN A MONTH... 3
- LESS THAN ONCE A MONTH...4
- NEVER...5
- DON'T KNOW... 9

24 How often does your husband/partner drink alcohol?

- EVERY DAY OR NEARLY EVERY DAY..1
- ONCE OR TWICE A WEEK...2
- 1-3 TIMES IN A MONTH...3
- LESS THAN ONCE A MONTH....4
- NEVER...5
- DON'T KNOW.....9

25 In the past 12 months how often have you seen your husband/partner drunk?

- MOST DAYS...1
- WEEKLY...2
- ONCE A MONTH......3
- LESS THAN ONCE A MONTH......4
- NEVER......5

26 How often does/did your husband/partner use hard drugs?

- EVERY DAY OR NEARLY EVERY DAY 1
- ONCE OR TWICE A WEEK.....2
- 1-3 TIMES IN A MONTH......3
- LESS THAN ONCE A MONTH... ...4
- NEVER...5
- DON'T KNOW... 9

27 Since you have known him, has he ever been involved in a physical fight with another man?

- YES... 1
- NO...2
- DON'T KNOW .. 9

28 In the past 12 months, how often has this happened?

- NEVER….1
- ONCE OR TWICE……2
- A FEW (3-5) TIMES….3
- DON'T KNOW……9

29 Has your husband/ partner had a relationship with any other women while being with you?

- YES...... 1
- NO… 2
- MAY HAVE… ... 3
- DON'T KNOW 9

30 Has your husband/partner had children with any other woman while being with you?

- YES…1
- NO… 2
- MAY HAVE… 3
- DON'T KNOW 9

31 As far as you know, was your partner's mother hit or beaten by her husband?

- YES.... 1
- NO 2
- PARENTS DID NOT LIVE TOGETHER. . 3
- DON'T KNOW 9

31B While growing up, was your mother hit or beaten by her husband

- YES....1
- NO 2
- PARENT DID NOT LIVE TOGETHER. ..3
- DIDN'T LIVE WITH PARENTS.... 4

32 As far as you know, was your husband/partner beaten regularly by any of his parents or members of his family while growing up?

- YES...... 1
- NO 2
- DON'T KNOW........ 9

32B While growing up, were you beaten regularly by your parents or members of your family

- YES......1
- NO 2
- DON'T KNOW 9

SECTION 5: ATITUDES TOWARDS GENDER ROLES

In this community and elsewhere, people have different ideas about families and what is acceptable behaviour for men and women in the home. I am going to read you a list of statements, and I would like you to tell me whether YOU generally agree or disagree with the statement. There are no right or wrong answers.

---

33 In your opinion, does a man have a good reason to hit his wife if she does not complete her household work to his satisfaction

- YES.........1
- NO......2

34 In your opinion, does a man have a good reason to hit his wife if She disobeys him

- YES... 1
- NO 2

35 In your opinion, does a man have a good reason to hit his wife if she refuses to have sexual relations with him

- YES... 1
- NO....... 2

36 In your opinion, does a man have a good reason to hit his wife if she asks him whether he has other girlfriends

- YES... 1
- NO ...2

37 In your opinion, does a man have a good reason to hit his wife if He suspects that the wife is unfaithful to him

- YES... 1
- NO...2

38 In your opinion, does a man have a good reason to hit his wife if He finds out that the wife is unfaithful to him

- YES.....1
- NO... 2

39 In your opinion, can a married woman refuse to have sex with her husband if She doesn't want to

- YES...1
- NO......2

40 In your opinion, can a married woman refuse to have sex with her husband if He is drunk

- YES......1
- NO...... 2

41 In your opinion, can a married woman refuse to have sex with her husband if She is sick

- YES...1
- NO...2

42 In your opinion, can a married woman refuse to have sex with her husband if He mistreats her

- YES...1
- NO... 2

SECTION 6: RESPONDENT AND HER PARTNER

When two people marry or live together, they usually share both good and bad moments. I would now like to ask you some questions about your current and past relationships and how your husband/partner treats (treated) you. If anyone interrupts us I will change the topic of conversation. I would again like to assure you that your answers will be kept secret. May I continue?

---

43 In general, do you and your husband/partner discuss the following topics together: Things that have happened to HIM in the day

- YES.........1
- NO... 2

44 HIS worries or feelings

- YES. 1
- NO 2

45 Things that happen to YOU during the day

- YES... 1
- NO..... 2

46 YOUR worries or feelings

- YES... 1
- NO..... 2

47 In your relationship with your husband/partner, how often would you say that you quarreled?

- RARELY... 1
- SOMETIMES... 2
- OFTEN 3

CONTROLLING BEHAVIOUR BEGINS

I am now going to ask you about some situations that are true for many women. Thinking about your husband/partner, would you say it is generally true that he

---

48 Prevent you from seeing friends

- ◯ YES....1
- ◯ NO......2

49 Restrict contact with your family of birth

- ◯ YES....1
- ◯ NO.....2

50 Insists on knowing where you are at all times

- ◯ YES....1
- ◯ NO... ...2

51 Ignores you or neglect you

- ◯ YES... .. 1
- ◯ NO... 2

52 Gets angry if you speak with another man

- ◯ YES... 1
- ◯ NO..... 2

53 Is often suspicious that you are unfaithful

- ◯ YES...1
- ◯ NO... 2

54 Expects you to ask his permission before seeking health care for yourself

- ◯ YES... .. 1
- ◯ NO..... 2

CONTROLLING BEHAVIOUR ENDS

---

55 Has any of these CONTROLLING BEHAVIOURS happened in the last 12 months

- ◯ YES...1
- ◯ NO...2

EMOTIONAL VL BEGINS

The next questions are about things that happen to many women, and that your partner, may have done to you. I want you to tell me if your partner, has ever done the following things to you:

---

56 Has he Ever Insulted you or called you names

IF NO TO QUESTION 57, PLEASE GO TO QUESTION 59

- YES...1
- NO......2

57 Has this happened in the last 12 months?

- YES...1
- NO....2

58 Has he Ever embarrassed you in public ?

IF NO TO QUESTION 59, PLEASE GO TO QUESTION 61

- YES...1
- NO...2

59 Has this happened in the last 12 months?

- YES...1
- NO...2

60 Has he Ever made you afraid e.g. yelling or looking at you in a particular way ?

IF NO TO QUESTION 61, PLEASE GO TO QUESTION 63

- YES...1
- NO...2

61 Has this happened in the last 12 months?

- YES.... 1
- NO..... 2

62 Has he Ever Threatened to hurt you ?

IF NO TO QUESTION 62, PLEASE GO TO QUESTION 64

- YES...1
- NO... 2

63 Has this happened in the last 12 months?

- YES...1
- NO...2

64 Do you ever feel nothing you do is good enough for your partner

I IF NO TO QUESTION TO 64 GO TO QUESTION 64C

- YES.... 1
- NO 2

64B Has this happened in the last 12 months?

- YES. 1
- NO 2

64C Has he ever sent you packing out of the house?

IF NO TO QUESTION 64C GO TO QUESTION 64E

- YES..... 1
- NO 2

64D Has this happened in the last 12 months

- YES... 1
- NO 2

64E Has he ever walked out of the house for at least a day

- YES..... 1
- NO 2

64F Has this happened in the last 12 months

- YES.... 1
- NO 2

EMOTIONAL VL ENDS

PHYSICAL VL BEGINS

The next questions are about things that happen to many women, and that your partner may have done to you. I want you to tell me if your current husband/partner, has ever done the following things to you.

---

65 Ever Slapped you?

IF NO TO QUESTION 65, PLEASE GO TO QUESTION 67

- YES.... 1
- NO... 2

66 Has this happened in the last 12 months?

- YES...1
- NO...2

67 Ever Pushed you or shoved you?

IF NO TO QUESTION 67, PLEASE GO TO QUESTION 69

- YES...1
- NO....2

68 Has this happened in the last 12 months?

- YES...1
- NO...2

69 Ever Hit you with open hand or fist?

IF NO TO QUESTION 69, PLEASE GO TO QUESTION 71

- YES...1
- NO....2

70 Has this happened in the last 12 months?

- YES...1
- NO...2

70A Ever hit you with something that could harm you?

- YES... 1
- NO 2

70B Has this happened in the last 12 month?

- YES.... 1
- NO 2

71 Ever beaten you up?

IF NO TO QUESTION 71, PLEASE GO TO QUESTION 73

- YES...1
- NO... 2

72 Has this happened in the last 12 months?

- YES...1
- NO...2

73 Ever Choked you?

IF NO TO QUESTION 73, PLEASE GO TO QUESTION 75

- YES...1
- NO......2

74 Has this happened in the last 12 months?

- YES...1
- NO....2

75 Ever Threatened to use or actually used a knife or other weapon against you?

IF NO TO QUESTION 75, PLEASE GO TO QUESTION 77

- YES...1
- NO...2

76 Has this happened in the last 12 months?

- YES.... 1
- NO ...2

PHYSICAL VL ENDS

SEXUAL VL BEGINS

THE NEXT QUESTIONS ARE THINGS THAT HAPPEN TO MANY WOMEN, AND MAY HAVE HAPPENED TO YOU, I WANT YOU TO TELL ME IF YOU HAVE EXPERIENCED THE FOLLOWING THINGS

HAS YOUR PARTNER

---

77 Ever forced you to have sexual intercourse when you did not want to?

IF NO TO QUESTION 77, PLEASE GO TO QUESTION 79

- YES......1
- NO....2

78 Has this happened in the last 12 months?

- YES......1
- NO......2

79 Did you ever have sexual intercourse when you did not want to because you were afraid ?

IF NO TO QUESTION 79, PLEASE GO TO QUESTION 81

- YES......1
- NO... ... 2

80 Has this happened in the last 12 months?

- YES......1
- NO......2

81 Have you ever been forced to perform degrading sexual act?

IF NO TO QUESTION 81, PLEASE GO TO QUESTION 83

- YES… 1
- NO… 2

82 Has this happened in the last 12 months?

- YES… 1
- NO… 2

SEXUAL VL ENDS

THERE ARE MANY THINGS THAT OUR HUSBANDS DO THAT AFFECT US IN SEVERAL WAYS. WE WANT TO ASK YOU QUESTIONS ABOUT THESE.

---

83 In what ways has the behavior of your husband/partner affected you? (READ OUT, TICK ALL THAT APPLY)

- [ ] Makes you feel sad/worried
- [ ] Makes you doubt the relationship
- [ ] Makes you feel less of yourself
- [ ] Makes you abuse alcohol
- [ ] Makes you use hard drugs
- [ ] Violence resulted in bruises/wound or other injuries
- [ ] Ever resulted in injury to the head
- [ ] Ever resulted in admission in the hospital
- [ ] Ever resulted in sight or hearing impairment
- [ ] Ever resulted in unplanned pregnancy
- [ ] Ever resulted in miscarriage or other
- [ ] Ever resulted in abortion
- [ ] Ever resulted in sexual transmitted infection
- [ ] Made you loose/quit your job
- [ ] Resulted in loss of tooth/broken bone
- [ ] Gives you suicidal thoughts
- [ ] None of the Above

84 How would you say that your partner's behavior has affected you

- ○ A little. 1
- ○ a lot........ 2

85 Are there any particular situations that tend to lead to violence?

- [ ] WAS NEVER VIOLENT...... 1
- [ ] WHEN MAN DRUNK........2
- [ ] MONEY PROBLEMS........3
- [ ] DIFFICULTIES AT HIS WORK....... 4
- [ ] WHEN HE IS UNEMPLOYED 5
- [ ] NO FOOD AT HOME........ 6
- [ ] PROBLEMS WITH HIS OR HER FAMILY 7
- [ ] SHE IS PREGNANT.......... 8
- [ ] HE IS JEALOUS OF HER......9
- [ ] SHE REFUSES SEX..........10
- [ ] SHE IS DISOBEDIENT 11
- [ ] OTHERS.......12

86 If others, please specify

____

87 In your life, how many times were you injured by your husband/partner

PLEASE IF NEVER TO QUESTION 91 PLEASE GO TO QUESTION 112

- ○ NEVER..... 1
- ○ ONCE/TWICE ...2
- ○ SEVERAL (3-5) TIMES.... 3
- ○ MANY (MORE THAN 5) TIMES... 4

88 Has this happened in the past 12 months?

- ○ YES...1
- ○ NO... 2

89 Were you ever hurt badly enough that you needed health care?

- ○ YES...1
- ○ NO... 2

90 For any of incident of physical violence, were your children present or did they overhear you being beaten?

- ○ HUSBAND WAS NEVER VIOLENT ..1
- ○ YES... 2
- ○ NO....3

91 Who have you told about the violence? READ OUT OPTIONS TICK ALL THAT APPLY

- [ ] WAS NEVER VIOLENT… 1
- [ ] NO ONE. .. 2
- [ ] FRIENDS. 3
- [ ] PARENT... 4
- [ ] BROTHER OR SISTER. ..5
- [ ] UNCLE OR AUTY... 6
- [ ] HUSB AND/PARTNER'S FAMILY .. 7
- [ ] CHILDREN. 8
- [ ] NEIGBOUR. . 9
- [ ] POLICE...10
- [ ] DOCTOR/HEALTH WORKER. 11
- [ ] COUNCELLOR. .. 12
- [ ] PASTOR/RELIGIOUS LEADER. 13
- [ ] NGO/WOMEN'S ORGANIZATION. 14
- [ ] GOVERNMENT ORGANIZATION...15
- [ ] LAWYER/COURT ...16
- [ ] SHELTER.... 17
- [ ] LOCAL LEADERS. . 18
- [ ] OTHERS... 19

92 If others, please specify

____

93 Did anyone ever try to help you? TICK ALL THAT APPLY

- [ ] HUSBAND WAS NEVER VIOLENT...1
- [ ] NO ONE...2
- [ ] FRIENDS. 3
- [ ] PARENT ...4
- [ ] BROTHER OR SISTER. ..5
- [ ] UNCLE OR AUTY..6
- [ ] HUSB AND/PARTNER'S FAMILY 6
- [ ] CHILDREN. 7
- [ ] NEHIBOUR. 8
- [ ] POLICE... 9
- [ ] DOCTOR/HEALTH WORKER....10
- [ ] PASTOR/RELIGIOUS LEADER. ... 12
- [ ] COUNCELLOR. 13
- [ ] NGO/WOMEN'S ORGANIZATION. ...14
- [ ] GOVERNMENT ORGANIZATION. 15
- [ ] LAWYER/COURT ... 16
- [ ] SHELTER... 17
- [ ] LOCAL LEADER. 18
- [ ] OTHERS:... 19

94 If others, please specify

____

95 What were the reasons that made you go for help? TICK ALL THAT APPLY

- [ ] HUSBAND NEVER VIOLENT .. 1
- [ ] I DID NOT GO FOR HELP ... 2
- [ ] ENCOURAGED BY FRIENDS/FAMILY 3
- [ ] COULD NOT ENDURE MORE. .. 4
- [ ] BADLY INJURED/AFRAID HE WOULD KILL HER. 5
- [ ] HE THREATENED OR TRIED TO KILL HER …5
- [ ] HE THREATENED OR HIT CHILDREN. ...6
- [ ] SAW THAT CHILDREN SUFFERING 7
- [ ] THROWN OUT OF THE HOME. 8
- [ ] AFRAID SHE WOULD KILL HIM… 9
- [ ] OTHERS. 9

96 If Others, please specify

____

97 Why didn't you go for help? TICK ALL THAT APPLY

PLEASE DO NOT READ OUT THE ANSWER OPTIONS LET THE RESPONDENTS SAY OUT THIER MIND AND TICK APPROPRIATELY

- [ ] WAS NEVER VIOLENT……1
- [ ] FEAR OF THREATS/CONSEQUENCES/MORE VIOLENCE….2
- [ ] VIOLENECE NORMAL/NOT SERIOUS….3
- [ ] EMBARRASSED/ASHAMED AFRAID WOULD NOT BE BELIEVED OR WOULD BE BLAMED…4
- [ ] BELIEVED NOT HELP/KNOW OTHER WOMEN NOT HELPED……5
- [ ] AFRAID WOULD END RELATIONSHIP…6
- [ ] AFRAID WOULD LOSE CHILDREN …7
- [ ] BRING BAD NAME TO FAMILY…8
- [ ] WAS NEVER VIOLENT... 9
- [ ] I WENT FOR HELP 10
- [ ] OTHERS. 11

98 If others, please specify

____

99 Is there anyone that you would like to receive (more) help from? Who? TICK ALL THAT APPLY

- [ ] WAS NEVER VIOLENT… 1
- [ ] FRIENDS. .. 2
- [ ] PARENTS. ... 3
- [ ] BROTHER OR SISTER. ...4
- [ ] UNCLE OR AUNT,. .. 5
- [ ] HUSB AND/PARTNER'S FAMILY ... 6
- [ ] CHILDREN. ... 7
- [ ] NEIGHBOURS. 8
- [ ] POLICE,. ..9
- [ ] DOCTOR/HEALTH WORKER. ... 10
- [ ] PASTOR/RELIGIOUS LEADER. 11
- [ ] COUNSELLOR. 12
- [ ] NGO/WOMEN'S ORGANIZATION. 13
- [ ] GOVERNMENT ORGANIZATION...... 14
- [ ] LAWYER/COURT ... 15
- [ ] SHELTER. ... 16
- [ ] LOCAL LEADER. 17
- [ ] NO ONE: 18
- [ ] OTHERS:.....19

100 If Others, please specify

____

101 Did you ever leave, even if overnight because of the violence

- ◯ YES.... 2
- ◯ NO.... 3

102 Where did you go? TICK ALL THAT APPLY

- [ ] HUSBAND NEVER VIOLENT ... 1
- [ ] I DID NOT LEAVE HOME.... 2
- [ ] HER RELATIVE....3
- [ ] HUSBAND RELATIVE.... 4
- [ ] HER FRIENDS/NEIGHBOURS. ..5
- [ ] HOTEL/LODGING. ... 6
- [ ] STREET... 7
- [ ] CHURCH/TEMPLE.....8
- [ ] SHELTER. 9
- [ ] OTHERS. .. 10

103 If others, please specify

---

104 On those occasions were you stayed, What were the reasons that made you stay

- [ ] HUSBAND WAS NEVER VIOLENT 1
- [ ] SITUATION WAS NOT SEVERE. 2
- [ ] DIDN'T W ANT TO LEAVE CHILDREN... 3
- [ ] SANCTITY OF MARRIAGE.... 4
- [ ] DIDN'T WANT TO BRING SHAME ON FAMILY... 5
- [ ] COULDN'T SUPPORT CHILDREN... 6
- [ ] LOVED HIM...7
- [ ] DIDN'T WANT TO BE SINGLE...8
- [ ] FAMILY SAID TO STAY ...9
- [ ] FORGAVE HIM...... 10
- [ ] THOUGHT HE WOULD CHANGE..... 11
- [ ] THREATENED HER CHILDREN...12
- [ ] NOWHERE TO GO...13
- [ ] OTHERS. 14

105 If others, please specify

SECTION 8 : FINANCIAL AUTONOMY

Now I would like to ask you some questions about things that you own and your earnings. We need this information to understand the financial position of women nowadays.

ECONOMIC VL BEGINS

---

106 Has your husband/partner EVER taken your earnings from you against your will?

F NO QUESTION 114, PLEASE GO TO QUESTION 116

- YES…….1
- NO… 2

107 Has this happened in the last 12 months?

- YES… 1
- NO…2

108 Does your husband/partner ever refuse to give you money for household expenses, even when he has money for other things?

I If NO TO QUESTION 117, PLEASE GO TO 119

- YES… 1
- NO… 2

109 Has this happened in the past 12 months ?

- YES…1
- NO…2

110 Do you suspect your husband may have stolen your money at some point

- YES.... 1
- NO 2

ECONOMIC VL ENDS

---

111 Who would you say earns more in the family?

- MORE THAN HUSBAND/PARTNER….1
- LESS THAN HUSBAND/PARTNER….2
- ABOUT THE SAME…3
- DO NOT KNOW… 9

112 In case of emergency, do you think that you alone could raise enough money to house and feed your family for 4 weeks?

- ◯ YES…..1
- ◯ NO… 2

113 In case of emergency, do you think that you alone could raise enough money to house and feed your family for 6 months?

- ◯ YES…..1
- ◯ NO….2

COMPLETION OF INTERVIEW

---

114 We have now finished the interview. Do you have any comments, or is there anything else you would like to add?

---

115 (NOT COMPULSORY) Would you like us to give a phone number where you can get help

- ◯ YES....1
- ◯ NO.... 2
