# Supplementary material for: Prevalence and determinants of emotional, physical, and sexual Intimate Partner Violence in urban Lagos, Nigeria: A cross-sectional study
Source: PLoS One. 2026 Oct 5;21(10):e0359973. doi: 10.1371/journal.pone.0359973 (PMC13638058; doi:10.1371/journal.pone.0359973)
Supplement: Supplementary file 2 [file pone.0359973.s004.pdf]

RESPONSE TO REVIEWERS

EDITOR COMMENTS:

1. Modify the title of the manuscript to include the study design

Response: Thank you for this suggestion. The title has been modified to include study design (Line 4-5)

2. Where does this work stand in body of knowledge concerning intimate partner violence in Lagos/Nigeria?

Response: Thank you for this important comment. We have revised the Introduction to better position this study within the existing body of knowledge on IPV in Nigeria. Specifically, we highlighted gaps in the literature, including the limited number of community-based studies in highly urbanised settings such as Lagos and the relative lack of studies examining emotional, physical, and sexual IPV concurrently. We have also clarified how this study contributes context-specific evidence from an urban LGA using a standardised WHO-based instrument, thereby enhancing comparability with existing national and international research. (Line 140 to 153)

3.Under study population, were the women still married/cohabiting at the time of study?

Response: Yes, they were. This has been corrected. (Line 197-201)

4.Research istrument: what was the cronbach's alpha score? how were the research assistants trained, by who and for how long? where was the pretest done?

Response: The assistants were trained by the researchers for one day. Pretest was done at Isolo LGA. This has been stated.(Line 241-243)

The WHO Multi-country Study questionnaire is a standardized and widely validated instrument developed for cross-cultural assessment of intimate partner violence. It comprises multiple domains rather than a single psychometric scale; therefore, a single Cronbach's alpha is not typically reported. Previous studies using the instrument have demonstrated good internal consistency for specific IPV domains, with Cronbach's alpha values generally ranging from approximately 0.65 to 0.90 depending on the subscale and study population. (Line 230-233)

5. Discussion - avoid repeating the the phrase "in our study/ in this study"

Response: Thank you for this observation. This has been corrected.

6. why was the Likert scale not used to assess atitude and how does your research instrument compare to the revised conflict tactic scale ?

The WHO Multi-Country Study on Women's Health and Domestic Violence questionnaire, which was used in this study, is a standardized and widely validated instrument specifically developed for measuring intimate partner violence (IPV) across diverse cultural settings. This instrument did not use Likert scale to assess attitude towards gender roles. Moreover,

behaviourally specific questions have been shown to improve recall and reduce misclassification compared to subjective attitudinal measures.

Regarding comparison with the Revised Conflict Tactics Scale (CTS2), both instruments are well-established tools for IPV research but differ in conceptual orientation and scope. The CTS2 focuses on conflict resolution tactics within relationships and includes subscales on negotiation, psychological aggression, physical assault, sexual coercion, and injury, often capturing bidirectional violence. In contrast, the WHO instrument was specifically designed for epidemiological studies of violence against women, with a stronger emphasis on women's lived experiences, contextual factors, and health consequences of IPV. It also incorporates modules on controlling behaviours and partner characteristics, which are particularly relevant for public health and gender-based violence research in LMIC settings.

Importantly, the WHO questionnaire has demonstrated good internal consistency in multiple settings and cross-cultural validity, making it appropriate for this study context. Its use aligns with global recommendations for IPV measurement in population-based surveys and enhances comparability with other studies conducted in similar settings.

REVIEWERS COMMENTS TO THE AUTHOR

Reviewer #1: Thank you for the opportunity to review the paper entitled: "Emotional, physical, and sexual intimate partner violence: prevalence and determinants in urban Lagos, Nigeria". This is a novel study whose originality lies mainly in the geographical and cultural context analysed, which has a remarkably large sample size and incorporates numerous variables related to the presence of IPV.
In my opinion, the manuscript would be greatly improved by incorporating the following suggestions:

First of all, I strongly recommend that the entire manuscript (text and tables) be reviewed in terms of spelling, punctuation marks, statistical symbols, and style, ensuring that everything complies with APA7 guidelines.

Response: Thank you very much for this comment. We have passed the work through Grammarly to improve the grammar.

The journal recommends Vancouver reference style.

1) Introduction:

1.1. Some of the ideas in the introduction are somewhat reductionist and the approach should be improved in order to properly contextualise IPV. For example, paragraph 2 (lines 82-83) states that women in developing countries are at greater risk of experiencing IPV due to "poor living conditions and social norms". The explanation should extend beyond living conditions from an economic standpoint, as this factor is not the one that most influences IPV. Furthermore, paragraph 4 (lines 107-108) states that 'children are left without the protection

of a mother', assuming that the focus is on women with young children. However, the approach should not be limited to this group of women with young children, as IPV affects women of all ages, with and without children. In any case, the wording could be amended to indicate that this research will indeed mention the existence of young children.

Response: Thank you for this comment. We agree that the initial phrasing was overly simplistic. The introduction has been revised to reflect the multifactorial nature of IPV. (Line 86-92)

We have revised the paragraph to emphasise that IPV affects women across the life course, regardless of parental status. (Line 125-132)

1.2. The Introduction should include some reasoning about the relevance of the selected independent variables.
Response:

Thank you for this comment. We have addressed this by providing justification for the selected independent variables in the Methods section (Data collection instruments and technique). Specifically, we clarified that the variables were guided by the WHO multi-country study framework and represent established individual, relational, and socio-economic determinants of IPV, ensuring consistency with existing evidence. (Line 150-153)

1.3. Please, revise if the fifth paragraph (lines 110-116) would better fit at the beginning of the Introduction.

Response: Thank you for this observation. It has been placed earlier to enhance flow. (Line 99-103)

2) Methods:

2.1. The second paragraph of the "Study area" section (lines 147-151) could be improved if the wording was more fluid. In addition, it would make it easier to understand if the authors explicitly provided some data on crime rates and socioeconomic class rather than simply saying that it is 'high'.

Response: Thank you for this helpful comment. We have revised the paragraph to improve clarity and flow. With regard to crime rates and socioeconomic status, we acknowledge the importance of providing quantitative data; however, reliable and up-to-date LGA-level statistics on crime rates and socioeconomic classification for Mushin were not readily available from publicly accessible sources. We have therefore rephrased the statement to adopt more neutral, evidence-consistent wording and retained appropriate references to support the general characterisation of the area, rather than making unsupported quantitative claims. (Line 184-190)

2.2. Could the symbol "/" be replaced with "and/or" in line 155: "Study design and study population" section?

Response: This has been rephrased better. (Line 197-200)

2.3. It is essential to incorporate in the "Methods" section the sample selection procedure, including how many women accepted, how many refused, the final sample size, etc.
Response: Thank you for this recommendation. This has been added. (Line 222-225)

2.4. I wonder how interviewers ensured the privacy of the women interviewed in their own homes with regard to their partners, whom they were reporting as perpetrators of IPV. It would be interesting to know the research approach in this regard and for the authors to explain whether the results may have been biased by this form of data collection.

Response: Thank you for this important comment. We recognise the sensitivity of collecting IPV data and the need to ensure participants' safety and confidentiality. During data collection, interviews were conducted in a private setting within the home, such as a secluded area where conversations could not be overheard. In many cases, interviews were conducted when partners were not present. Interviewers were trained in ethical guidelines for IPV research, including ensuring confidentiality, building rapport, and prioritising participant safety.

2.5. It would be useful to know whether the instruments used have been validated and, if so, to provide information on their psychometric properties (reliability, validity, cultural adaptation, etc.) and the sources that support their use in this context.

Response: Thank you for this important comment. The study utilised the WHO Multi-country Study on Women's Health and Domestic Violence questionnaire, a widely used and standardised instrument that has been validated across multiple cultural settings, including low- and middle-income countries. The tool has demonstrated good reliability and validity in measuring different forms of IPV and is based on behaviourally specific items, which are considered the gold standard for IPV research. It has also been extensively used in sub-Saharan Africa and Nigeria. (Line 230-233)

3) Results:

3.1. Please, include the standard deviation (SD) measure in all required cases.

Response: Thank you for your comment. The SD is written with the means ±SD

3.2. In the first paragraph, it would make the text more coherent if the sentence about income (lines 278-279) was placed after the one about the type of employment of women.

Response: Thank you. This has been done. (Line 335-336)

3.3. Again, with regard to the presence of children, I would recommend to explain (in the

Methods section and in the Results section) whether respondents were only asked about minor children or whether adult sons and daughters were also taken into account, bearing in mind that the age of women varies from 20 to "over 40" (in this case, it would also be relevant to know the age of the oldest woman).

Response: The questionnaire asks: How many children do you have? The questionnaire does not specify whether minor children or adult sons or daughters. The questionnaire is provided in the Additional files.

3.4. In page 11, lines 319-321, the percentages given for each of the two variables explained do not correspond to those shown in Table 2, so please, correct this mistake.

Response: Thank you for drawing our attention to this. It has been corrected. (Line 387-388)

4) Discussion and conclusions:

4.1. IPV stems from individual, social, and structural factors that go beyond socio-economic stress and social disorder, so I believe it would be advisable to qualify this statement (lines 548-550).

Response: Thank you for this insightful comment. We have revised the discussion to better reflect the multifactorial nature of IPV, acknowledging the interplay of individual, relational, community, and structural determinants, rather than attributing it primarily to socioeconomic stress and social disorder. (Line 624-629)

4.2. In paragraph 5 (lines 566-573), the explanation of violence in terms of the length of the relationship leaves out some other elements that could be interacting with that greater experience in more recent relationships. These might include, among others, the age of women and their perception and capacity to identify and verbalise the different types of violence they are suffering (bearing in mind that people in shorter relationships are likely to be younger). Furthermore, the reference 24 is a study from the 1990s, a time when the information available nowadays about IPV, 30 years later, was not as accessible or visible as it is today.

Response: Thank you for this insightful comment. We have revised the discussion to provide a more nuanced interpretation of the association between duration of union and IPV, incorporating potential confounding and interacting factors such as age, cohort effects, and differences in awareness and reporting. We have also tempered reliance on older literature and emphasised that the relationship is multifactorial and should be interpreted with caution. (Line 646-659)

4.3. Something similar happens with paragraph 7 (lines 585-595). In addition to the explanation about social status based on the number of children, this fact might also be linked to the length of the relationship (in shorter relationships, it has not yet been possible to have more children, and raising the first child can be a significant stressor that leads to IPV). So, I

wonder if the reference to the studies 28 and 29 are the most appropriate for contrasting this variable.

Response: Thank you for this insightful comment. We have revised the discussion to provide a more nuanced interpretation of the association between number of children and IPV, incorporating the potential influence of relationship duration, transition to parenthood, and related stressors. We have also moderated the emphasis on social status and clarified that the relationship is multifactorial and context-dependent.(Line 682-692)

5) Implications for policy and practice:

5.1. It would be advisable to examine the practical implications in greater detail, as it may be complex to propose specific interventions aimed at high-risk groups in relation to infidelity, for example.

Response: Thank you for this important comment. We have revised the implications section to more explicitly link the study findings to practical and implementable interventions. Specifically, we expanded the discussion to reflect the role of relationship dynamics, partner behaviours (e.g., alcohol use, aggression), and intergenerational factors, and reframed recommendations towards feasible strategies such as male engagement, couple-based interventions, and health-system strengthening, rather than focusing on isolated risk factors. (Line 772-797)

REVIEWER #2:

The article is timely and actually reflect the realities in Nigeria. Is well-written and should be published. However, There is need of grammar checks across the article especially in the result section were the authors lumps some results together making the reader struggling to understand the meaning. The initial 3 paragraphs of the discussion section did not obey the standard discussion format, that needs to be reviewed. Finally, a lot of figure were quoted in the discussion section, those figures are not necessary and can be reduced to only where necessary.

Response: We thank the reviewer for the kind words.

The manuscript's grammar has been improved with the use of Grammarly software and changes have also been made in the results section.

The discussion now starts by stating the study objectives. (line 592-594). The discussion then summarises the main findings before discussing in subsequent paragraphs.
