# Supplementary material for: Prevalence and determinants of emotional, physical, and sexual Intimate Partner Violence in urban Lagos, Nigeria: A cross-sectional study
Source: PLoS One. 2026 Oct 5;21(10):e0359973. doi: 10.1371/journal.pone.0359973 (PMC13638058; doi:10.1371/journal.pone.0359973)
Supplement: Supplementary file 3 [file pone.0359973.s002.docx]

**Table S1: Bivariate analysis showing associations between selected variables and Emotional IPV in the preceding 12 months**

| **Variables** | **Experienced Emotional IPV** | **Did not experience Emotional IPV** | **p-value** |
| --- | --- | --- | --- |
|  | **Freq(%)** | **Freq(%)** |  |
| **Age (in years)** |  |  |  |
| 20-29 | 35(38.9) | 55(61.1) | <0.001 |
| 30-39 | 99(44.8) | 122(55.2) |  |
| ≥40 | 128(67) | 63(33) |  |
| **Marital status** |  |  |  |
| Legally married | 190(62.9) | 112(37.1) | <0.001 |
| Co-habiting | 72(36) | 128(64) |  |
| Number of years in union |  |  |  |
| 2 - 10 | 111(41.9) | 154(58.1) | <0.001 |
| 11-20 | 85(55.9) | 67(44.1) |  |
| > 20 | 66(77.6) | 19(22.4) |  |
| **Type of marriage** |  |  |  |
| Monogamous | 204(53.4) | 178(46.6) | 0.332 |
| Polygamous | 58(48.3) | 62(51.7) |  |
| **Highest level of education** |  |  |  |
| No formal education | 12(75) | 4(25) | 0.292 |
| Primary education | 45(53.6) | 39(46.4) |  |
| Secondary education | 166(51.4) | 157(48.6) |  |
| Post secondary education | 39(49.4) | 40(50.6) |  |
| **Occupation** |  |  |  |
| Unemployed | 11(61.1) | 7(38.9) | 0.179 |
| Unskilled/Manual | 105(47.1) | 118(52.9) |  |
| Semi-skilled | 140(56.5) | 108(43.5) |  |
| Skilled | 6(46.2) | 7(53.8) |  |
| **Religion** |  |  |  |
| Christianity | 129(52) | 119(48) | 0.938 |
| Islam | 133(52.4) | 121(47.6) |  |
| **Ethnicity** |  |  |  |
| Yoruba | 207(48.6) | 219(51.4) | <0.001 |
| non-Yoruba | 55(72.4) | 21(27.6) |  |
| **Monthly income** |  |  |  |
| <N20,000 (< $48.19) | 46(48.9) | 48(51.1) | 0.16 |
| N20,000 – N39,999 ($48.19-$96.38) | 152(50.3) | 150(49.7) |  |
| >N40,000 (≥$96.39) | 64(60.4) | 42(39.6) |  |
| **Number of children** |  |  |  |
| 1 | 33(36.3) | 58(63.7) | <0.001 |
| 2-3 | 123(46.9) | 139(53.1) |  |
| ≥4 | 106(71.1) | 43(28.9) |  |
| **Partner’s age (in years)** |  |  |  |
| <35 | 19(31.7) | 41(68.3) | <0.001 |
| 35-45 | 100(42.4) | 136(57.6) |  |
| >45 | 143(69.4) | 63(30.6) |  |
| **Partner’s highest level of education** |  |  |  |
| No formal education | 5(50) | 5(50) | 0.631 |
| Primary education | 43(58.9) | 30(41.1) |  |
| Secondary education | 159(50.5) | 156(49.5) |  |
| Higher | 55(52.9) | 49(47.1) |  |
| **Partner’s occupation** |  |  |  |
| Unemployed/unskilled | 26(57.8) | 19(42.2) | 0.432 |
| Semi-skilled/skilled | 236(51.6) | 221(48.4) |  |
| **Tobacco or smoking history of partner** |  |  |  |
| Smokes/use tobacco | 21(27.6) | 55(72.4) | <0.001 |
| Does not smoke/use tobacco | 241(56.6) | 185(43.4) |  |
| **Alcohol drinking history of partner** |  |  |  |
| Everyday/nearly everyday | 50(36) | 89(64) | <0.001 |
| Once or twice a week | 58(49.2) | 60(50.8) |  |
| Never/less than once a week | 154(62.9) | 91(37.1) |  |
| **Partner use of hard drugs** |  |  |  |
| Uses hard drugs | 8(19) | 34(81) | <0.001 |
| Does not use hard drugs | 254(55.2) | 206(44.8) |  |
| **Partner involved in physical fight with another man** |  |  |  |
| No | 232(58) | 168(42) | <0.001 |
| Yes | 30(29.4) | 72(70.6) |  |
| **Partner having extra-marital relationship** |  |  |  |
| No | 161(61.7) | 100(38.3) | <0.001 |
| Yes | 67(38.5) | 107(61.5) |  |
| Don’t know | 34(50.7) | 33(49.3) |  |
| **Partner had children outside wedlock during marriage** |  |  |  |
| No | 227(53.9) | 194(46.1) | 0.077 |
| Yes | 35(43.2) | 46(56.8) |  |
| **History of partner’s mother being beaten by her husband** |  |  |  |
| No | 163(57.8) | 119(42.2) | 0.015 |
| Yes | 10(50) | 10(50) |  |
| Don’t know | 89(44.5) | 111(55.5) |  |
| **Partner was beaten regularly by parents or members of his family while growing up** |  |  |  |
| No | 110(52.9) | 98(47.1) | 0.794 |
| Yes | 152(51.7) | 142(48.3) |  |
| **Communication with partner** |  |  |  |
| Poor communication | 21(26.2) | 59(73.8) | <0.001 |
| Good communication | 241(57.1) | 181(42.9) |  |
| **Attitude towards gender roles** |  |  |  |
| Poor attitude | 3(42.9) | 4(57.1) | 0.619 |
| Good attitude | 259(52.3) | 236(47.7) |  |

**Table S2: Bivariate analysis showing associations between selected variables and Physical IPV in the preceding 12 months**

| **Variables** | **Experienced Physical IPV** | **Did not experience Physical IPV** | **p-value** |
| --- | --- | --- | --- |
|  | **Freq(%)** | **Freq(%)** |  |
| **Age (in years)** |  |  |  |
| 20-29 | 54(60) | 36(40) | <0.001 |
| 30-39 | 146(66.1) | 75(33.9) |  |
| ≥40 | 164(85.9) | 27(14.1) |  |
| **Marital status** |  |  |  |
| Legally married | 252(83.4) | 50(16.6) | <0.001 |
| Co-habiting | 112(56) | 88(44) |  |
| **Number of years in union** |  |  |  |
| 2-10 | 172(64.9) | 93(35.1) | <0.001 |
| 11-20 | 115(75.7) | 37(24.3) |  |
| > 20 | 77(90.6) | 8(9.4) |  |
| **Type of marriage** |  |  |  |
| Monogamous | 274(71.7) | 108(28.3) | 0.484 |
| Polygamous | 90(75) | 30(25) |  |
| **Highest level of education** |  |  |  |
| No formal education | 14(87.5) | 2(12.5) | 0.246 |
| Primary education | 56(66.7) | 28(33.3) |  |
| Secondary education | 233(72.1) | 90(27.9) |  |
| Post secondary education | 61(77.2) | 18(22.8) |  |
| **Occupation** |  |  |  |
| Unemployed | 13(72.2) | 5(27.8) | 0.382 |
| Unskilled/Manual | 154(69.1) | 69(30.9) |  |
| Semi-skilled | 186(75) | 62(25) |  |
| Skilled | 11(84.6) | 2(15.4) |  |
| **Religion** |  |  |  |
| Christianity | 169(68.1) | 79(31.9) | 0.03 |
| Islam | 195(76.8) | 59(23.2) |  |
| **Ethnicity** |  |  |  |
| Yoruba | 302(70.9) | 124(29.1) | 0.055 |
| non-Yoruba | 62(81.6) | 14(18.4) |  |
| **Monthly income** |  |  |  |
| <N20,000 (< $48.19) | 61(64.9) | 33(35.1) | 0.034 |
| N20,000 – N39,999 ($48.19-$96.38) | 217(71.9) | 85(28.1) |  |
| >N40,000 (≥$96.39) | 86(81.1) | 20(18.9) |  |
| **Number of children** |  |  |  |
| 1 | 51(56) | 40(44) | <0.001 |
| 2-3 | 181(69.1) | 81(30.9) |  |
| ≥4 | 132(88.6) | 17(11.4) |  |
| **Partner’s age (in years)** |  |  |  |
| <35 | 31(51.7) | 29(48.3) | <0.001 |
| 35-45 | 156(66.1) | 80(33.9) |  |
| >45 | 177(85.9) | 29(14.1) |  |
| **Partner’s highest level of education** |  |  |  |
| No formal education | 8(80) | 2(20) | 0.208 |
| Primary education | 54(74) | 19(26) |  |
| Secondary education | 219(69.5) | 96(30.5) |  |
| Higher | 83(79.8) | 21(20.2) |  |
| **Partner’s occupation** |  |  |  |
| Unemployed/unskilled | 32(71.1) | 13(28.9) | 0.826 |
| Semi-skilled/skilled | 332(72.6) | 125(27.4) |  |
| Tobacco or smoking history of partner |  |  |  |
| Smokes/use tobacco | 36(47.4) | 40(52.6) | <0.001 |
| Does not smoke/use tobacco | 328(77) | 98(23) |  |
| **Alcohol drinking history of partner** |  |  |  |
| Everyday/nearly everyday | 74(53.2) | 65(46.8) | <0.001 |
| Once or twice a week | 83(70.3) | 35(29.7) |  |
| Never/less than once a week | 207(84.5) | 38(15.5) |  |
| **Partner use of hard drugs** |  |  |  |
| Uses hard drugs | 13(31) | 29(69) | <0.001 |
| Does not use hard drugs | 351(76.3) | 109(23.7) |  |
| **Partner involved in physical fight with another man** |  |  |  |
| No | 321(80.2) | 79(19.8) | <0.001 |
| Yes | 43(42.2) | 59(57.8) |  |
| **Partner having extra-marital relationship** |  |  |  |
| No | 210(80.5) | 51(19.5) | <0.001 |
| Yes | 105(60.3) | 69(39.7) |  |
| Don’t know | 49(73.1) | 18(26.9) |  |
| **Partner had children outside wedlock during marriage** |  |  |  |
| No | 306(72.7) | 115(27.3) | 0.842 |
| Yes | 58(71.6) | 23(28.4) |  |
| **History of partner’s mother being beaten by her husband** |  |  |  |
| No | 231(81.9) | 51(18.1) | <0.001 |
| Yes | 10(50) | 10(50) |  |
| Don’t know | 123(61.5) | 77(38.5) |  |
| **Partner was beaten regularly by parents or members of his family while growing up** |  |  |  |
| No | 151(72.6) | 57(27.4) | 0.971 |
| Yes | 213(72.4) | 81(27.6) |  |
| **Communication with partner** |  |  |  |
| Poor communication | 34(42.5) | 46(57.5) | <0.001 |
| Good communication | 330(78.2) | 92(21.8) |  |
| **Attitude towards gender roles** |  |  |  |
| Poor attitude | 4(57.1) | 3(42.9) | 0.359 |
| Good attitude | 360(72.7) | 135(27.3) |  |

**Table S3: Bivariate analysis showing associations between selected variables and Sexual IPV in the preceding 12 months**

| **Variables** | **Experienced Physical IPV** | **Did not experience Physical IPV** | **p-value** |
| --- | --- | --- | --- |
|  | **Freq(%)** | **Freq(%)** |  |
| **Age (in years)** |  |  |  |
| 20-29 | 54(60) | 36(40) | <0.001 |
| 30-39 | 146(66.1) | 75(33.9) |  |
| ≥40 | 164(85.9) | 27(14.1) |  |
| **Marital status** |  |  |  |
| Legally married | 252(83.4) | 50(16.6) | <0.001 |
| Co-habiting | 112(56) | 88(44) |  |
| **Number of years in union** |  |  |  |
| 2-10 | 172(64.9) | 93(35.1) | <0.001 |
| 11-20 | 115(75.7) | 37(24.3) |  |
| > 20 | 77(90.6) | 8(9.4) |  |
| **Type of marriage** |  |  |  |
| Monogamous | 274(71.7) | 108(28.3) | 0.484 |
| Polygamous | 90(75) | 30(25) |  |
| **Highest level of education** |  |  |  |
| No formal education | 14(87.5) | 2(12.5) | 0.246 |
| Primary education | 56(66.7) | 28(33.3) |  |
| Secondary education | 233(72.1) | 90(27.9) |  |
| Post secondary education | 61(77.2) | 18(22.8) |  |
| **Occupation** |  |  |  |
| Unemployed | 13(72.2) | 5(27.8) | 0.382 |
| Unskilled/Manual | 154(69.1) | 69(30.9) |  |
| Semi-skilled | 186(75) | 62(25) |  |
| Skilled | 11(84.6) | 2(15.4) |  |
| **Religion** |  |  |  |
| Christianity | 169(68.1) | 79(31.9) | 0.03 |
| Islam | 195(76.8) | 59(23.2) |  |
| **Ethnicity** |  |  |  |
| Yoruba | 302(70.9) | 124(29.1) | 0.055 |
| non-Yoruba | 62(81.6) | 14(18.4) |  |
| **Monthly income** |  |  |  |
| <N20,000 (< $48.19) | 61(64.9) | 33(35.1) | 0.034 |
| N20,000 – N39,999 ($48.19-$96.38) | 217(71.9) | 85(28.1) |  |
| >N40,000 (≥$96.39) | 86(81.1) | 20(18.9) |  |
| **Number of children** |  |  |  |
| 1 | 51(56) | 40(44) | <0.001 |
| 02-3 | 181(69.1) | 81(30.9) |  |
| ≥4 | 132(88.6) | 17(11.4) |  |
| **Partner’s age (in years)** |  |  |  |
| <35 | 31(51.7) | 29(48.3) | <0.001 |
| 35-45 | 156(66.1) | 80(33.9) |  |
| >45 | 177(85.9) | 29(14.1) |  |
| **Partner’s highest level of education** |  |  |  |
| No formal education | 8(80) | 2(20) | 0.208 |
| Primary education | 54(74) | 19(26) |  |
| Secondary education | 219(69.5) | 96(30.5) |  |
| Higher | 83(79.8) | 21(20.2) |  |
| **Partner’s occupation** |  |  |  |
| Unemployed/unskilled | 32(71.1) | 13(28.9) | 0.826 |
| Semi-skilled/skilled | 332(72.6) | 125(27.4) |  |
| **Tobacco or smoking history of partner** |  |  |  |
| Smokes/use tobacco | 36(47.4) | 40(52.6) | <0.001 |
| Does not smoke/use tobacco | 328(77) | 98(23) |  |
| **Alcohol drinking history of partner** |  |  |  |
| Everyday/nearly everyday | 74(53.2) | 65(46.8) | <0.001 |
| Once or twice a week | 83(70.3) | 35(29.7) |  |
| Never/less than once a week | 207(84.5) | 38(15.5) |  |
| **Partner use of hard drugs** |  |  |  |
| Uses hard drugs | 13(31) | 29(69) | <0.001 |
| Does not use hard drugs | 351(76.3) | 109(23.7) |  |
| **Partner involved in physical fight with another man** |  |  |  |
| No | 321(80.2) | 79(19.8) | <0.001 |
| Yes | 43(42.2) | 59(57.8) |  |
| **Partner having extra-marital relationship** |  |  |  |
| No | 210(80.5) | 51(19.5) | <0.001 |
| Yes | 105(60.3) | 69(39.7) |  |
| Don’t know | 49(73.1) | 18(26.9) |  |
| **Partner had children outside wedlock during marriage** |  |  |  |
| No | 306(72.7) | 115(27.3) | 0.842 |
| Yes | 58(71.6) | 23(28.4) |  |
| **History of partner’s mother being beaten by her husband** |  |  |  |
| No | 231(81.9) | 51(18.1) | <0.001 |
| Yes | 10(50) | 10(50) |  |
| Don’t know | 123(61.5) | 77(38.5) |  |
| **Partner was beaten regularly by parents or members of his family while growing up** |  |  |  |
| No | 151(72.6) | 57(27.4) | 0.971 |
| Yes | 213(72.4) | 81(27.6) |  |
| **Communication with partner** |  |  |  |
| Poor communication | 34(42.5) | 46(57.5) | <0.001 |
| Good communication | 330(78.2) | 92(21.8) |  |
| **Attitude towards gender roles** |  |  |  |
| Poor attitude | 4(57.1) | 3(42.9) | 0.359 |
| Good attitude | 360(72.7) | 135(27.3) |  |

**Table S4: Bivariate analysis showing associations between selected variables and Any IPV in the preceding 12 months**

|  | **Experienced Any IPV** | **Did not experience Any IPV** | **p-value** |
| --- | --- | --- | --- |
|  | **Freq(%)** | **Freq(%)** |  |
| **Age (in years)** |  |  |  |
| 20-29 | 29(32.2) | 61(67.8) | <0.001 |
| 30-39 | 91(41.2) | 130(58.8) |  |
| ≥40 | 122(63.9) | 69(36.1) |  |
| **Marital status** |  |  |  |
| Legally married | 181(59.9) | 121(40.1) | <0.001 |
| Co-habiting | 61(30.5) | 139(69.5) |  |
| **Number of years in union** |  |  |  |
| 2-10 | 100(37.7) | 165(62.3) | <0.001 |
| 11-20 | 79(52) | 73(48) |  |
| > 20 | 63(74.1) | 22(25.9) |  |
| **Type of marriage** |  |  |  |
| Monogamous | 185(48.4) | 197(51.6) | 0.859 |
| Polygamous | 57(47.5) | 63(52.5) |  |
| **Highest level of education** |  |  |  |
| No formal education education | 12(75) | 4(25) | 0.165 |
| Primary education | 42(50) | 42(50) |  |
| Secondary education | 152(47.1) | 171(52.9) |  |
| Post secondary education | 36(45.6) | 43(54.4) |  |
| **Occupation** |  |  |  |
| Unemployed | 9(50) | 9(50) | 0.285 |
| Unskilled/Manual | 97(43.5) | 126(56.5) |  |
| Semi-skilled | 130(52.4) | 118(47.6) |  |
| Skilled | 6(46.2) | 7(53.8) |  |
| **Religion** |  |  |  |
| Christianity | 116(46.8) | 132(53.2) | 0.525 |
| Islam | 126(49.6) | 128(50.4) |  |
| **Ethnicity** |  |  |  |
| Yoruba | 189(44.4) | 237(55.6) | <0.001 |
| non-Yoruba | 53(69.7) | 23(30.3) |  |
| **Monthly income** |  |  |  |
| <N20,000 (< $48.19) | 41(43.6) | 53(56.4) | 0.086 |
| N20,000 – N39,999 ($48.19-$96.38) | 140(46.4) | 162(53.6) |  |
| >N40,000 (≥$96.39) | 61(57.5) | 45(42.5) |  |
| **Number of children** |  |  |  |
| 1 | 26(28.6) | 65(71.4) | <0.001 |
| 2-3 | 116(44.3) | 146(55.7) |  |
| ≥4 | 100(67.1) | 49(32.9) |  |
| **Partner’s age (in years)** |  |  |  |
| <35 | 15(25) | 45(75) | <0.001 |
| 35-45 | 90(38.1) | 146(61.9) |  |
| >45 | 137(66.5) | 69(33.5) |  |
| **Partner’s highest level of education** |  |  |  |
| No formal education | 5(50) | 5(50) | 0.385 |
| Primary education | 42(57.5) | 31(42.5) |  |
| Secondary | 146(46.3) | 169(53.7) |  |
| Higher | 49(47.1) | 55(52.9) |  |
| **Partner’s occupation** |  |  |  |
| Unemployed/unskilled | 25(55.6) | 20(44.4) | 0.301 |
| Semi-skilled/skilled | 217(47.5) | 240(52.5) |  |
| **Tobacco or smoking history of partner** |  |  |  |
| Smokes/use tobacco | 19(25) | 57(75) | <0.001 |
| Does not smoke/use tobacco | 223(52.3) | 203(47.7) |  |
| **Alcohol drinking history of partner** |  |  |  |
| Everyday/nearly everyday | 48(34.5) | 91(65.5) | <0.001 |
| Once or twice a week | 52(44.1) | 66(55.9) |  |
| Never/less than once a week | 142(58) | 103(42) |  |
| **Partner use of hard drugs** |  |  |  |
| Uses hard drugs | 7(16.7) | 35(83.3) | <0.001 |
| Does not use hard drugs | 235(51.1) | 225(48.9) |  |
| **Partner involved in physical fight with another man** |  |  |  |
| No | 220(55) | 180(45) | <0.001 |
| Yes | 22(21.6) | 80(78.4) |  |
| **Partner having extra-marital relationship** |  |  |  |
| No | 151(57.9) | 110(42.1) | <0.001 |
| Yes | 60(34.5) | 114(65.5) |  |
| Don’t know | 31(46.3) | 36(53.7) |  |
| **Partner had children outside wedlock during marriage** |  |  |  |
| No | 208(49.4) | 213(50.6) | 0.22 |
| Yes | 34(42) | 47(58) |  |
| **History of partner’s mother being beaten by her husband** |  |  |  |
| No | 153(54.3) | 129(45.7) | 0.008 |
| Yes | 7(35) | 13(65) |  |
| Don’t know | 82(41) | 118(59) |  |
| **Partner was beaten regularly by parents or members of his family while growing up** |  |  |  |
| No | 103(49.5) | 105(50.5) | 0.621 |
| Yes | 139(47.3) | 155(52.7) |  |
| **Communication with partner** |  |  |  |
| Poor communication | 20(25) | 60(75) | <0.001 |
| Good communication | 222(52.6) | 200(47.4) |  |
| **Attitude towards gender roles** |  |  |  |
| Poor attitude | 1(14.3) | 6(85.7) | 0.07 |
| Good attitude | 241(48.7) | 254(51.3) |  |
