# Supplementary material for: Prevalence and determinants of emotional, physical, and sexual Intimate Partner Violence in urban Lagos, Nigeria: A cross-sectional study
Source: PLoS One. 2026 Oct 5;21(10):e0359973. doi: 10.1371/journal.pone.0359973 (PMC13638058; doi:10.1371/journal.pone.0359973)
Supplement: Supplementary file 4 [file pone.0359973.s005.docx]

**RESPONSE TO REVIEWERSTop of Form**

**PONE-D-25-58389R1**

**Prevalence and determinants of emotional, physical, and sexual Intimate Partner Violence in urban Lagos, Nigeria: a cross-sectional study**

PLOS One
Journal Requirements:

1. If the reviewer comments include a recommendation to cite specific previously published works, please review and evaluate these publications to determine whether they are relevant and should be cited. There is no requirement to cite these works unless the editor has indicated otherwise. 

2. Please review your reference list to ensure that it is complete and correct. If you have cited papers that have been retracted, please include the rationale for doing so in the manuscript text, or remove these references and replace them with relevant current references. Any changes to the reference list should be mentioned in the rebuttal letter that accompanies your revised manuscript. If you need to cite a retracted article, indicate the article’s retracted status in the References list and also include a citation and full reference for the retraction notice.
**Response:** Thank you for this comment. We have gone through the list again and it is accurate.

**Reviewers' comments:**

Please use the space provided to explain your answers to the questions above. You may also include additional comments for the author, including concerns about dual publication, research ethics, or publication ethics. (Please upload your review as an attachment if it exceeds 20,000 characters)

Reviewer #1: Please, review the writing style throughout the manuscript again to avoid repetitions and to ensure a fluent reading.

Response: Thank you for this comment. We have reviewed the manuscript again and made some changes in the introduction to limit repetitions (Line 79-102)

Reviewer #3: The research is community based and there is need to comply with National Health Research Ethics Committee of Nigeria Code of health research ethics by involving the Community before, during and after conduct of the research. If that has been done, then the researchers need to state it clearly under the section for ethical considerations.
All other comments raised during the preview have been addressed.

**Response:** Thank you for this important observation. Relevant community leaders and representatives were engaged before the study, supported community entry during data collection, and were informed of the findings after the study. This information has now been added to the “Ethics approval and consent to participate” section (Line 334-336).

Reviewer #4: Thank you for the opportunity to review this manuscript, which addresses an important public health issue by examining the prevalence and determinants of emotional, physical, and sexual intimate partner violence (IPV) among women in urban Lagos, Nigeria. The study is relevant to ongoing efforts to understand the burden and drivers of IPV in low- and middle-income settings and has the potential to contribute valuable evidence to inform policy and intervention strategies. The manuscript is generally well structured and the research question is of public health significance. However, several issues related to the clarity, methodological reporting, internal consistency, and presentation of results require attention before the manuscript can be considered for publication. My specific comments are provided below.
Abstract:
i. Indicate how the participants were selected (i.e., the sampling technique) in the Methods section of the abstract.
**Response:** Thank you for this comment. We have included the sampling technique in the abstract (Line 43).

Introduction
i. The second and third paragraphs are conceptually repetitive, as both discuss gender inequality, unequal power relations, and patriarchal norms as underlying drivers of intimate partner violence. Consider integrating these concepts into a single paragraph to eliminate redundancy, improve the flow of the introduction, and provide a smoother transition to the subsequent discussion of the burden of IPV in Nigeria.

**Response:** Thank you for this comment. We have modified these two paragraphs to remove repetition and redundancy (Line 79-102).

Study area
i. Consider including the literacy or educational profile of the study area, as this is an important contextual factor relevant to intimate partner violence.
ii. Please provide a brief justification for the selection of Mushin LGA as the study site by highlighting characteristics that make it relevant to the study of intimate partner violence. This will strengthen the contextual basis of the study.

**Response:** Thank you for this helpful comment. The Study Area section has been revised to include the educational and literacy profile of Mushin LGA. We have also clarified that Mushin has a high population density, relatively low socioeconomic status, educational challenges, diverse population and predominance of informal economic activities (Line 153-177).

Sample size
i. The rationale for inflating the sample size by 30% for non-response requires further justification. Given the interviewer-administered data collection approach, this assumption appears relatively high unless supported by evidence from previous studies, the unique logistical and ethical challenges associated with IPV research, etc.

**Response to reviewer**

Thank you for this observation. The 30% allowance was a conservative estimate made during the study-planning stage because the sensitive nature of IPV questions and the privacy requirements for household interviews were expected to result in refusals or incomplete interviews. However, the actual response rate was 90.6%, which was higher than anticipated. We have revised the Sample Size Determination section (Line 193-197)

Sampling methods
The multistage sampling procedure requires further clarification to ensure reproducibility. Specifically, please indicate
i. the total number of streets included in the sampling frame
ii. the number selected from each ward, together with the basis for determining this number;
iii. the procedure used to select one eligible woman within a selected household when more than one woman met the inclusion criteria;
iv. clarify the selection process in houses containing multiple households by distinguishing household selection from respondent selection.

**Response:** Thank you for highlighting the need for greater clarity regarding the multistage sampling procedure. Data collection was conducted in 2022, and unfortunately, the field sampling records are no longer available to verify the exact number of streets in the sampling frame, the number selected from each ward, the number of houses listed, or the basis for determining the number selected per ward. We therefore cannot provide these figures reliably and have avoided retrospectively introducing unverifiable information.

However, we have clarified that one household was selected by balloting in houses with multiple households and, where necessary, one eligible woman was similarly selected from that household(Line 205-212).

Data collection instruments and techniques
I. First, the statement that the WHO multi-country questionnaire has been "widely validated across diverse settings" is too general. Please specify the settings in which the instrument has been validated (e.g., LMICs, African countries, or settings comparable to Lagos) and provide the relevant supporting references.
II. Second, indicate the language(s) in which the interviews were conducted
III. Third, the statement that "privacy was ensured during the interviews" should be expanded to describe the specific measures taken to ensure privacy in the household setting, given the sensitive nature of IPV research.
IV. Revise the description of the pretest for clarity by stating that the questionnaire was pretested among participants in Isolo LGA, explaining the rationale for selecting the pretest site

**Response:** Thank you for these helpful comments. We have specified that the WHO questionnaire was developed and implemented in diverse low- and middle-income settings, including Ethiopia, Namibia and Tanzania, with the supporting reference provided. We have also stated that interviews were conducted in English or Yoruba, described the measures used to maintain privacy, and clarified that the questionnaire was pretested among eligible women in Isolo LGA because it provided also, an urban setting (Line 226-231).

Results
The Results section is not well aligned with the study objectives. Tables 3 and 4 give disproportionate attention to attitudes towards gender roles and partner communication, which are explanatory variables rather than the primary outcomes. In contrast, emotional, physical, and sexual IPV (the main outcomes of the study) are presented only as composite prevalence estimates in Table 5.
i. Since these outcomes were assessed using multiple questionnaire items, present the individual items (or a summary of them) before the composite prevalence estimates to show how the outcomes were derived.
ii. If attitudes and partner communication are not central to the study objectives, reduce their prominence in the descriptive results and focus on them in the analysis of factors associated with IPV. This will improve the coherence and alignment of the Results section with the study objectives and title.
iii. Please verify the frequencies and percentages reported for the overall attitude towards gender roles in Table 3. The reported values appear inconsistent with the study sample size (n = 502), as the frequencies do not sum to the total sample and the percentage reported for the negative attitude category does not correspond to its frequency.
 **Response:** Thank you for this important observation. We have revised the Results section to improve its alignment with the study objectives and to give greater prominence to emotional, physical, and sexual intimate partner violence, which are the primary outcomes of the study.

i. Table 5 has been expanded to present the individual questionnaire items used to assess emotional, physical, and sexual IPV. For each item, the frequency and percentage of respondents reporting the experience during their lifetime and within the preceding 12 months are now shown. The accompanying narrative highlights the most commonly reported acts within each IPV domain. The composite prevalence estimates for emotional, physical, sexual, and any IPV are presented after the item-level findings, thereby showing more clearly how the overall IPV outcomes were derived.

ii. We agree that attitudes towards gender roles and partner communication are explanatory variables rather than the primary study outcomes. We have therefore reduced their emphasis in the accompanying narrative and clarified that they were assessed as potential determinants of IPV. The tables have been retained because both variables were included in the subsequent analysis of factors associated with IPV, and their descriptive distributions provide context for interpreting those findings. However, the revised Results section now places greater narrative emphasis on the item-level and composite IPV findings.

iii. We rechecked the analysis for overall attitude towards gender roles and identified that the frequencies previously entered in Table 3 were incorrect. The corrected results show that 7 respondents (1.4%) had a negative attitude, while 495 respondents (98.6%) had a positive attitude towards gender roles. These frequencies sum to the total sample of 502, and the corresponding percentages sum to 100.0%. Table 3 and the accompanying narrative have been corrected accordingly.

(Line 338-656)

Limitations
i. Please indicate the measures taken to mitigate recall and social desirability biases.

**Response:** Thank you for this comment. We have revised the Limitations section to state the measures used to minimise recall and social desirability biases, including trained female interviewers, private interviews, assurances of confidentiality, behaviourally specific questions, and clearly defined reference periods (Line 801-805).
