# Supplementary material for: Prevalence and determinants of emotional, physical, and sexual Intimate Partner Violence in urban Lagos, Nigeria: A cross-sectional study
Source: PLoS One. 2026 Oct 5;21(10):e0359973. doi: 10.1371/journal.pone.0359973 (PMC13638058; doi:10.1371/journal.pone.0359973)
Supplement: Peer review history

# Peer review: Decision Letter 0

**DOI:** 10.1371/journal.pone.0359973.r001

**Authors:** Adewale Ashimi

24 Mar 2026

Dear Dr.  Olubodun,

Thank you for submitting your manuscript to PLOS ONE. After careful consideration, we feel that it has merit but does not fully meet PLOS ONE’s publication criteria as it currently stands. Therefore, we invite you to submit a revised version of the manuscript that addresses the points raised during the review process.

Please submit your revised manuscript by May 08 2026 11:59PM. If you will need more time than this to complete your revisions, please reply to this message or contact the journal office at plosone@plos.org. When you're ready to submit your revision, log on to https://www.editorialmanager.com/pone/ and select the 'Submissions Needing Revision' folder to locate your manuscript file.

- A letter that responds to each point raised by the academic editor and reviewer(s). You should upload this letter as a separate file labeled 'Response to Reviewers'.
- A marked-up copy of your manuscript that highlights changes made to the original version. You should upload this as a separate file labeled 'Revised Manuscript with Track Changes'.
- An unmarked version of your revised paper without tracked changes. You should upload this as a separate file labeled 'Manuscript'.

If you would like to make changes to your financial disclosure, please include your updated statement in your cover letter. Guidelines for resubmitting your figure files are available below the reviewer comments at the end of this letter.

If applicable, we recommend that you deposit your laboratory protocols in protocols.io to enhance the reproducibility of your results. Protocols.io assigns your protocol its own identifier (DOI) so that it can be cited independently in the future. For instructions see: https://journals.plos.org/plosone/s/submission-guidelines#loc-laboratory-protocols. Additionally, PLOS ONE offers an option for publishing peer-reviewed Lab Protocol articles, which describe protocols hosted on protocols.io. Read more information on sharing protocols at https://plos.org/protocols?utm_medium=editorial-email&utm_source=authorletters&utm_campaign=protocols.

We look forward to receiving your revised manuscript.

Kind regards,

Adewale Olufemi Ashimi, MBBS, MPH, PhD, FWACS

Academic Editor

PLOS One

Journal Requirements:

When submitting your revision, we need you to address these additional requirements.

1.Please ensure that your manuscript meets PLOS ONE's style requirements, including those for file naming. The PLOS ONE style templates can be found at

https://journals.plos.org/plosone/s/file?id=wjVg/PLOSOne_formatting_sample_main_body.pdf and

https://journals.plos.org/plosone/s/file?id=ba62/PLOSOne_formatting_sample_title_authors_affiliations.pdf

2. Please include captions for your Supporting Information files at the end of your manuscript, and update any in-text citations to match accordingly. Please see our Supporting Information guidelines for more information: http://journals.plos.org/plosone/s/supporting-information.

3. If the reviewer comments include a recommendation to cite specific previously published works, please review and evaluate these publications to determine whether they are relevant and should be cited. There is no requirement to cite these works unless the editor has indicated otherwise.

Additional Editor Comments:

1. Modify the title of the manuscript to include the study design

2. where dooes this work stand in body of knowledge concerning intimate partner violence in lagos/Nigeria?

3.Under study population, were the women still married/cohabiting at the time of study?

4.Research istrument: what was the cronbach's alpha score? how were the research assistants trained, by who and for how long? where was the pretest done?

5. Discussion - avoid repeating the the phrase "in our study/ in this study"

why was the Likert scale not used to assess atitude and how does your research instrument compare to the revised conflict tactic scale ?

[Note: HTML markup is below. Please do not edit.]

Reviewer's Responses to Questions

**Comments to the Author**

1. Is the manuscript technically sound, and do the data support the conclusions?

Reviewer #1: Partly

Reviewer #2: Yes

**********

2. Has the statistical analysis been performed appropriately and rigorously?

Reviewer #1: Yes

Reviewer #2: Yes

**********

3. Have the authors made all data underlying the findings in their manuscript fully available?

The PLOS Data policy

Reviewer #1: Yes

Reviewer #2: Yes

**********

4. Is the manuscript presented in an intelligible fashion and written in standard English?

Reviewer #1: No

Reviewer #2: Yes

**********

Reviewer #1: Thank you for the opportunity to review the paper entitled: “Emotional, physical, and sexual intimate partner violence: prevalence and determinants in urban Lagos, Nigeria”. This is a novel study whose originality lies mainly in the geographical and cultural context analysed, which has a remarkably large sample size and incorporates numerous variables related to the presence of IPV.

In my opinion, the manuscript would be greatly improved by incorporating the following suggestions:

First of all, I strongly recommend that the entire manuscript (text and tables) be reviewed in terms of spelling, punctuation marks, statistical symbols, and style, ensuring that everything complies with APA7 guidelines.

1) Introduction:

1.1. Some of the ideas in the introduction are somewhat reductionist and the approach should be improved in order to properly contextualise IPV. For example, paragraph 2 (lines 82-83) states that women in developing countries are at greater risk of experiencing IPV due to "poor living conditions and social norms". The explanation should extend beyond living conditions from an economic standpoint, as this factor is not the one that most influences IPV. Furthermore, paragraph 4 (lines 107-108) states that ‘children are left without the protection of a mother’, assuming that the focus is on women with young children. However, the approach should not be limited to this group of women with young children, as IPV affects women of all ages, with and without children. In any case, the wording could be amended to indicate that this research will indeed mention the existence of young children.

1.2. The Introduction should include some reasoning about the relevance of the selected independent variables.

1.3. Please, revise if the fifth paragraph (lines 110-116) would better fit at the beginning of the Introduction.

2) Methods:

2.1. The second paragraph of the “Study area” section (lines 147-151) could be improved if the wording was more fluid. In addition, it would make it easier to understand if the authors explicitly provided some data on crime rates and socioeconomic class rather than simply saying that it is ‘high’.

2.2. Could the symbol “/” be replaced with “and/or” in line 155: “Study design and study population” section?

2.3. It is essential to incorporate in the “Methods” section the sample selection procedure, including how many women accepted, how many refused, the final sample size, etc.

2.4. I wonder how interviewers ensured the privacy of the women interviewed in their own homes with regard to their partners, whom they were reporting as perpetrators of IPV. It would be interesting to know the research approach in this regard and for the authors to explain whether the results may have been biased by this form of data collection.

2.5. It would be useful to know whether the instruments used have been validated and, if so, to provide information on their psychometric properties (reliability, validity, cultural adaptation, etc.) and the sources that support their use in this context.

3) Results:

3.1. Please, include the standard deviation (SD) measure in all required cases.

3.2. In the first paragraph, it would make the text more coherent if the sentence about income (lines 278-279) was placed after the one about the type of employment of women.

3.3. Again, with regard to the presence of children, I would recommend to explain (in the Methods section and in the Results section) whether respondents were only asked about minor children or whether adult sons and daughters were also taken into account, bearing in mind that the age of women varies from 20 to “over 40” (in this case, it would also be relevant to know the age of the oldest woman).

3.4. In page 11, lines 319-321, the percentages given for each of the two variables explained do not correspond to those shown in Table 2, so please, correct this mistake.

3.5. Authors should try not to duplicate the data that appears in tables and in the text. The text should flow naturally, and the tables should be clear, so that a simple reference in the text allows the reader to go to the table to check the corresponding data. It is not necessary for all the data to appear in both places.

4) Discussion and conclusions:

4.1. IPV stems from individual, social, and structural factors that go beyond socio-economic stress and social disorder, so I believe it would be advisable to qualify this statement (lines 548-550).

4.2. In paragraph 5 (lines 566-573), the explanation of violence in terms of the length of the relationship leaves out some other elements that could be interacting with that greater experience in more recent relationships. These might include, among others, the age of women and their perception and capacity to identify and verbalise the different types of violence they are suffering (bearing in mind that people in shorter relationships are likely to be younger). Furthermore, the reference 24 is a study from the 1990s, a time when the information available nowadays about IPV, 30 years later, was not as accessible or visible as it is today.

4.3. Something similar happens with paragraph 7 (lines 585-595). In addition to the explanation about social status based on the number of children, this fact might also be linked to the length of the relationship (in shorter relationships, it has not yet been possible to have more children, and raising the first child can be a significant stressor that leads to IPV). So, I wonder if the reference to the studies 28 and 29 are the most appropriate for contrasting this variable.

5) Implications for policy and practice:

5.1. It would be advisable to examine the practical implications in greater detail, as it may be complex to propose specific interventions aimed at high-risk groups in relation to infidelity, for example.

Reviewer #2: The article is timely and actually reflect the realities in Nigeria. Is well-written and should be published. However, There is need of grammar checks across the article especially in the result section were the authors lumps some results together making the reader struggling to understand the meaning. The initial 3 paragraphs of the discussion section did not obey the standard discussion format, that needs to be reviewed. Finally, a lot of figure were quoted in the discussion section, those figures are not necessary and can be reduced to only where necessary.

**********

what does this mean?). If published, this will include your full peer review and any attached files.

If you choose “no”, your identity will remain anonymous but your review may still be made public.

**Do you want your identity to be public for this peer review?**  For information about this choice, including consent withdrawal, please see our Privacy Policy

Reviewer #1: No

Reviewer #2: **Yes:**  Abdulrahman Ahmad

**********

[NOTE: If reviewer comments were submitted as an attachment file, they will be attached to this email and accessible via the submission site. Please log into your account, locate the manuscript record, and check for the action link "View Attachments". If this link does not appear, there are no attachment files.]

To ensure your figures meet our technical requirements, please review our figure guidelines: https://journals.plos.org/plosone/s/figures

You may also use PLOS’s free figure tool, NAAS, to help you prepare publication quality figures: https://journals.plos.org/plosone/s/figures#loc-tools-for-figure-preparation.

NAAS will assess whether your figures meet our technical requirements by comparing each figure against our figure specifications.

# Author comment: Author response to Decision Letter 1

**DOI:** 10.1371/journal.pone.0359973.r002

4 May 2026

RESPONSE TO EDITOR AND REVIEWERS

EDITOR COMMENTS:

1. Modify the title of the manuscript to include the study design

Response: Thank you for this suggestion. The title has been modified to include study design (Line 4-5)

2. Where does this work stand in body of knowledge concerning intimate partner violence in Lagos/Nigeria?

Response: Thank you for this important comment. We have revised the Introduction to better position this study within the existing body of knowledge on IPV in Nigeria. Specifically, we highlighted gaps in the literature, including the limited number of community-based studies in highly urbanised settings such as Lagos and the relative lack of studies examining emotional, physical, and sexual IPV concurrently. We have also clarified how this study contributes context-specific evidence from an urban LGA using a standardised WHO-based instrument, thereby enhancing comparability with existing national and international research. (Line 140 to 153)

3.Under study population, were the women still married/cohabiting at the time of study?

Response: Yes, they were. This has been corrected. (Line 197-201)

4.Research istrument: what was the cronbach's alpha score? how were the research assistants trained, by who and for how long? where was the pretest done?

Response: The assistants were trained by the researchers for one day. Pretest was done at Isolo LGA. This has been stated.( Line 241-243)

The WHO Multi-country Study questionnaire is a standardized and widely validated instrument developed for cross-cultural assessment of intimate partner violence. It comprises multiple domains rather than a single psychometric scale; therefore, a single Cronbach’s alpha is not typically reported. Previous studies using the instrument have demonstrated good internal consistency for specific IPV domains, with Cronbach’s alpha values generally ranging from approximately 0.65 to 0.90 depending on the subscale and study population. (Line 230-233)

5. Discussion - avoid repeating the the phrase "in our study/ in this study"

Response: Thank you for this observation. This has been corrected.

6. why was the Likert scale not used to assess atitude and how does your research instrument compare to the revised conflict tactic scale ?

The WHO Multi-Country Study on Women’s Health and Domestic Violence questionnaire, which was used in this study, is a standardized and widely validated instrument specifically developed for measuring intimate partner violence (IPV) across diverse cultural settings. This instrument did not use Likert scale to assess attitude towards gender roles. Moreover, behaviourally specific questions have been shown to improve recall and reduce misclassification compared to subjective attitudinal measures.

Regarding comparison with the Revised Conflict Tactics Scale (CTS2), both instruments are well-established tools for IPV research but differ in conceptual orientation and scope. The CTS2 focuses on conflict resolution tactics within relationships and includes subscales on negotiation, psychological aggression, physical assault, sexual coercion, and injury, often capturing bidirectional violence. In contrast, the WHO instrument was specifically designed for epidemiological studies of violence against women, with a stronger emphasis on women’s lived experiences, contextual factors, and health consequences of IPV. It also incorporates modules on controlling behaviours and partner characteristics, which are particularly relevant for public health and gender-based violence research in LMIC settings.

Importantly, the WHO questionnaire has demonstrated good internal consistency in multiple settings and cross-cultural validity, making it appropriate for this study context. Its use aligns with global recommendations for IPV measurement in population-based surveys and enhances comparability with other studies conducted in similar settings.

REVIEWERS COMMENTS TO THE AUTHOR

Reviewer #1: Thank you for the opportunity to review the paper entitled: “Emotional, physical, and sexual intimate partner violence: prevalence and determinants in urban Lagos, Nigeria”. This is a novel study whose originality lies mainly in the geographical and cultural context analysed, which has a remarkably large sample size and incorporates numerous variables related to the presence of IPV.

In my opinion, the manuscript would be greatly improved by incorporating the following suggestions:

First of all, I strongly recommend that the entire manuscript (text and tables) be reviewed in terms of spelling, punctuation marks, statistical symbols, and style, ensuring that everything complies with APA7 guidelines.

Response: Thank you very much for this comment. We have passed the work through Grammarly to improve the grammar.

The journal recommends Vancouver reference style.

1) Introduction:

1.1. Some of the ideas in the introduction are somewhat reductionist and the approach should be improved in order to properly contextualise IPV. For example, paragraph 2 (lines 82-83) states that women in developing countries are at greater risk of experiencing IPV due to "poor living conditions and social norms". The explanation should extend beyond living conditions from an economic standpoint, as this factor is not the one that most influences IPV. Furthermore, paragraph 4 (lines 107-108) states that ‘children are left without the protection of a mother’, assuming that the focus is on women with young children. However, the approach should not be limited to this group of women with young children, as IPV affects women of all ages, with and without children. In any case, the wording could be amended to indicate that this research will indeed mention the existence of young children.

Response: Thank you for this comment. We agree that the initial phrasing was overly simplistic. The introduction has been revised to reflect the multifactorial nature of IPV. (Line 86-92)

We have revised the paragraph to emphasise that IPV affects women across the life course, regardless of parental status. (Line 125-132)

1.2. The Introduction should include some reasoning about the relevance of the selected independent variables.

Response:

Thank you for this comment. We have addressed this by providing justification for the selected independent variables in the Methods section (Data collection instruments and technique). Specifically, we clarified that the variables were guided by the WHO multi-country study framework and represent established individual, relational, and socio-economic determinants of IPV, ensuring consistency with existing evidence. (Line 150-153)

1.3. Please, revise if the fifth paragraph (lines 110-116) would better fit at the beginning of the Introduction.

Response: Thank you for this observation. It has been placed earlier to enhance flow. (Line 99-103)

2) Methods:

2.1. The second paragraph of the “Study area” section (lines 147-151) could be improved if the wording was more fluid. In addition, it would make it easier to understand if the authors explicitly provided some data on crime rates and socioeconomic class rather than simply saying that it is ‘high’.

Response: Thank you for this helpful comment. We have revised the paragraph to improve clarity and flow. With regard to crime rates and socioeconomic status, we acknowledge the importance of providing quantitative data; however, reliable and up-to-date LGA-level statistics on crime rates and socioeconomic classification for Mushin were not readily available from publicly accessible sources. We have therefore rephrased the statement to adopt more neutral, evidence-consistent wording and retained appropriate references to support the general characterisation of the area, rather than making unsupported quantitative claims. (Line 184-190)

2.2. Could the symbol “/” be replaced with “and/or” in line 155: “Study design and study population” section?

Response: This has been rephrased better. (Line 197-200)

2.3. It is essential to incorporate in the “Methods” section the sample selection procedure, including how many women accepted, how many refused, the final sample size, etc.

Response: Thank you for this recommendation. This has been added. (Line 222-225)

2.4. I wonder how interviewers ensured the privacy of the women interviewed in their own homes with regard to their partners, whom they were reporting as perpetrators of IPV. It would be interesting to know the research approach in this regard and for the authors to explain whether the results may have been biased by this form of data collection.

Response: Thank you for this important comment. We recognise the sensitivity of collecting IPV data and the need to ensure participants’ safety and confidentiality. During data collection, interviews were conducted in a private setting within the home, such as a secluded area where conversations could not be overheard. In many cases, interviews were conducted when partners were not present. Interviewers were trained in ethical guidelines for IPV research, including ensuring confidentiality, building rapport, and prioritising participant safety.

2.5. It would be useful to know whether the instruments used have been validated and, if so, to provide information on their psychometric properties (reliability, validity, cultural adaptation, etc.) and the sources that support their use in this context.

Response: Thank you for this important comment. The study utilised the WHO Multi-country Study on Women’s Health and Domestic Violence questionnaire, a widely used and standardised instrument that has been validated across multiple cultural settings, including low- and middle-income countries. The tool has demonstrated good reliability and validity in measuring different forms of IPV and is based on behaviourally specific items, which are considered the gold standard for IPV research. It has also been extensively used in sub-Saharan Africa and Nigeria. (Line 230-233)

3) Results:

3.1. Please, include the standard deviation (SD) measure in all required cases.

Response: Thank you for your comment. The SD is written with the means ±SD

3.2. In the first paragraph, it would make the text more coherent if the sentence about income (lines 278-279) was placed after the one about the type of employment of women.

Response: Thank you. This has been done. (Line 335-336)

3.3. Again, with regard to the presence of children, I would recommend to explain (in the Methods section and in the Results section) whether respondents were only asked about minor children or whether adult sons and daughters were also taken into account, bearing in mind that the age of women varies from 20 to “over 40” (in this case, it would also be relevant to know the age of the oldest woman).

Response: The questionnaire asks: How many children do you have? The questionnaire does not specify whether minor children or adult sons or daughters. The questionnaire is provided in the Additional files.

3.4. In page 11, lines 319-321, the percentages given for each of the two variables explained do not correspond to those shown in Table 2, so please, correct this mistake.

Response: Thank you for drawing our attention to this. It has been corrected. (Line 387-388)

4) Discussion and conclusions:

4.1. IPV stems from individual, social, and structural factors that go beyond socio-economic stress and social disorder, so I believe it would be advisable to qualify this statement (lines 548-550).

Response: Thank you for this insightful comment. We have revised the discussion to better reflect the multifactorial nature of IPV, acknowledging the interplay of individual, relational, community, and structural determinants, rather than attributing it primarily to socioeconomic stress and social disorder. (Line 624-629)

4.2. In paragraph 5 (lines 566-573), the explanation of violence in terms of the length of the relationship leaves out some other elements that could be interacting with that greater experience in more recent relationships. These might include, among others, the age of women and their perception and capacity to identify and verbalise the different types of violence they are suffering (bearing in mind that people in shorter relationships are likely to be younger). Furthermore, the reference 24 is a study from the 1990s, a time when the information available nowadays about IPV, 30 years later, was not as accessible or visible as it is today.

Response: Thank you for this insightful comment. We have revised the discussion to provide a more nuanced interpretation of the association between duration of union and IPV, incorporating potential confounding and interacting factors such as age, cohort effects, and differences in awareness and reporting. We have also tempered reliance on older literature and emphasised that the relationship is multifactorial and should be interpreted with caution. (Line 646-659)

4.3. Something similar happens with paragraph 7 (lines 585-595). In addition to the explanation about social status based on the number of children, this fact might also be linked to the length of the relationship (in shorter relationships, it has not yet been possible to have more children, and raising the first child can be a significant stressor that leads to IPV). So, I wonder if the reference to the studies 28 and 29 are the most appropriate for contrasting this variable.

Response: Thank you for this insightful comment. We have revised the discussion to provide a more nuanced interpretation of the association between number of children and IPV, incorporating the potential influence of relationship duration, transition to parenthood, and related stressors. We have also moderated the emphasis on social status and clarified that the relationship is multifactorial and context-dependent.(Line 682-692)

5) Implications for policy and practice:

5.1. It would be advisable to examine the practical implications in greater detail, as it may be complex to propose specific interventions aimed at high-risk groups in relation to infidelity, for example.

Response: Thank you for this important comment. We have revised the implications section to more explicitly link the study findings to practical and implementable interventions. Specifically, we expanded the discussion to reflect the role of relationship dynamics, partner behaviours (e.g., alcohol use, aggression), and intergenerational factors, and reframed recommendations towards feasible strategies such as male engagement, couple-based interventions, and health-system strengthening, rather than focusing on isolated risk factors. (Line 772-797)

REVIEWER #2:

The article is timely and actually reflect the realities in Nigeria. Is well-written and should be published. However, There is need of grammar checks across the article especially in the result section were the authors lumps some results together making the reader struggling to understand the meaning. The initial 3 paragraphs of the discussion section did not obey the standard discussion format, that needs to be reviewed. Finally, a lot of figure were quoted in the discussion section, those figures are not necessary and can be reduced to only where necessary.

Response: We thank the reviewer for the kind words.

The manuscript’s grammar has been improved with the use of Grammarly software and changes have also been made in the results section.

The discussion now starts by stating the study objectives. (line 592-594). The discussion then summarises the main findings before discussing in subsequent paragraphs.

Attachment Submitted filename: Response to Reviewers.pdf

# Peer review: Decision Letter 1

**DOI:** 10.1371/journal.pone.0359973.r003

**Authors:** Adewale Ashimi

31 Jul 2026

Dear Dr. Olubodun,

Thank you for submitting your manuscript to PLOS ONE. After careful consideration, we feel that it has merit but does not fully meet PLOS ONE’s publication criteria as it currently stands. Therefore, we invite you to submit a revised version of the manuscript that addresses the points raised during the review process.

Please submit your revised manuscript by Sep 14 2026 11:59PM. If you will need more time than this to complete your revisions, please reply to this message or contact the journal office at plosone@plos.org. When you're ready to submit your revision, log on to https://www.editorialmanager.com/pone/ and select the 'Submissions Needing Revision' folder to locate your manuscript file.

- A letter that responds to each point raised by the academic editor and reviewer(s). You should upload this letter as a separate file labeled 'Response to Reviewers'.
- A marked-up copy of your manuscript that highlights changes made to the original version. You should upload this as a separate file labeled 'Revised Manuscript with Track Changes'.
- An unmarked version of your revised paper without tracked changes. You should upload this as a separate file labeled 'Manuscript'.

If you would like to make changes to your financial disclosure, please include your updated statement in your cover letter. Guidelines for resubmitting your figure files are available below the reviewer comments at the end of this letter.

If applicable, we recommend that you deposit your laboratory protocols in protocols.io to enhance the reproducibility of your results. Protocols.io assigns your protocol its own identifier (DOI) so that it can be cited independently in the future. For instructions see: https://journals.plos.org/plosone/s/submission-guidelines#loc-laboratory-protocols. Additionally, PLOS ONE offers an option for publishing peer-reviewed Lab Protocol articles, which describe protocols hosted on protocols.io. Read more information on sharing protocols at https://plos.org/protocols?utm_medium=editorial-email&utm_source=authorletters&utm_campaign=protocols.

As the corresponding author, your ORCID iD is verified in the submission system and will appear in the published article. PLOS supports the use of ORCID, and we encourage all coauthors to register for an ORCID iD and use it as well. Please encourage your coauthors to verify their ORCID iD within the submission system before final acceptance, as unverified ORCID iDs will not appear in the published article. *Only*  the individual author can complete the verification step; PLOS staff *cannot*  verify ORCID iDs on behalf of authors.

We look forward to receiving your revised manuscript.

Kind regards,

Adewale Olufemi Ashimi, MBChB, MPH, PhD, FWACS

Academic Editor

PLOS One

Journal Requirements:

1. If the reviewer comments include a recommendation to cite specific previously published works, please review and evaluate these publications to determine whether they are relevant and should be cited. There is no requirement to cite these works unless the editor has indicated otherwise.

2. Please review your reference list to ensure that it is complete and correct. If you have cited papers that have been retracted, please include the rationale for doing so in the manuscript text, or remove these references and replace them with relevant current references. Any changes to the reference list should be mentioned in the rebuttal letter that accompanies your revised manuscript. If you need to cite a retracted article, indicate the article’s retracted status in the References list and also include a citation and full reference for the retraction notice.

[Note: HTML markup is below. Please do not edit.]

Reviewers' comments:

Reviewer's Responses to Questions

**Comments to the Author**

Reviewer #1: All comments have been addressed

Reviewer #3: All comments have been addressed

Reviewer #4: (No Response)

**********

2. Is the manuscript technically sound, and do the data support the conclusions?

Reviewer #1: Yes

Reviewer #3: Yes

Reviewer #4: Partly

**********

3. Has the statistical analysis been performed appropriately and rigorously?

Reviewer #1: Yes

Reviewer #3: Yes

Reviewer #4: No

**********

4. Have the authors made all data underlying the findings in their manuscript fully available?

The PLOS Data policy

Reviewer #1: Yes

Reviewer #3: Yes

Reviewer #4: (No Response)

**********

5. Is the manuscript presented in an intelligible fashion and written in standard English?

Reviewer #1: Yes

Reviewer #3: Yes

Reviewer #4: Yes

**********

Reviewer #1: Please, review the writing style throughout the manuscript again to avoid repetitions and to ensure a fluent reading.

Reviewer #3: The research is community based and there is need to comply with National Health Research Ethics Committee of Nigeria Code of health research ethics by involving the Community before, during and after conduct of the research. If that has been done, then the researchers need to state it clearly under the section for ethical considerations.

All other comments raised during the preview have been addressed.

Reviewer #4: Thank you for the opportunity to review this manuscript, which addresses an important public health issue by examining the prevalence and determinants of emotional, physical, and sexual intimate partner violence (IPV) among women in urban Lagos, Nigeria. The study is relevant to ongoing efforts to understand the burden and drivers of IPV in low- and middle-income settings and has the potential to contribute valuable evidence to inform policy and intervention strategies. The manuscript is generally well structured and the research question is of public health significance. However, several issues related to the clarity, methodological reporting, internal consistency, and presentation of results require attention before the manuscript can be considered for publication. My specific comments are provided below.

Abstract:

i. Indicate how the participants were selected (i.e., the sampling technique) in the Methods section of the abstract.

Introduction

i. The second and third paragraphs are conceptually repetitive, as both discuss gender inequality, unequal power relations, and patriarchal norms as underlying drivers of intimate partner violence. Consider integrating these concepts into a single paragraph to eliminate redundancy, improve the flow of the introduction, and provide a smoother transition to the subsequent discussion of the burden of IPV in Nigeria.

Study area

i. Consider including the literacy or educational profile of the study area, as this is an important contextual factor relevant to intimate partner violence.

ii. Please provide a brief justification for the selection of Mushin LGA as the study site by highlighting characteristics that make it relevant to the study of intimate partner violence. This will strengthen the contextual basis of the study.

Sample size

i. The rationale for inflating the sample size by 30% for non-response requires further justification. Given the interviewer-administered data collection approach, this assumption appears relatively high unless supported by evidence from previous studies, the unique logistical and ethical challenges associated with IPV research, etc.

Sampling methods

The multistage sampling procedure requires further clarification to ensure reproducibility. Specifically, please indicate

i. the total number of streets included in the sampling frame

ii. the number selected from each ward, together with the basis for determining this number;

iii. the procedure used to select one eligible woman within a selected household when more than one woman met the inclusion criteria;

iv. clarify the selection process in houses containing multiple households by distinguishing household selection from respondent selection.

Data collection instruments and techniques

I. First, the statement that the WHO multi-country questionnaire has been "widely validated across diverse settings" is too general. Please specify the settings in which the instrument has been validated (e.g., LMICs, African countries, or settings comparable to Lagos) and provide the relevant supporting references.

II. Second, indicate the language(s) in which the interviews were conducted

III. Third, the statement that "privacy was ensured during the interviews" should be expanded to describe the specific measures taken to ensure privacy in the household setting, given the sensitive nature of IPV research.

IV. Revise the description of the pretest for clarity by stating that the questionnaire was pretested among participants in Isolo LGA, explaining the rationale for selecting the pretest site

Results

The Results section is not well aligned with the study objectives. Tables 3 and 4 give disproportionate attention to attitudes towards gender roles and partner communication, which are explanatory variables rather than the primary outcomes. In contrast, emotional, physical, and sexual IPV (the main outcomes of the study) are presented only as composite prevalence estimates in Table 5.

i. Since these outcomes were assessed using multiple questionnaire items, present the individual items (or a summary of them) before the composite prevalence estimates to show how the outcomes were derived.

ii. If attitudes and partner communication are not central to the study objectives, reduce their prominence in the descriptive results and focus on them in the analysis of factors associated with IPV. This will improve the coherence and alignment of the Results section with the study objectives and title.

iii. Please verify the frequencies and percentages reported for the overall attitude towards gender roles in Table 3. The reported values appear inconsistent with the study sample size (n = 502), as the frequencies do not sum to the total sample and the percentage reported for the negative attitude category does not correspond to its frequency.

Limitations

i. Please indicate the measures taken to mitigate recall and social desirability biases.

**********

what does this mean?). If published, this will include your full peer review and any attached files.

If you choose “no”, your identity will remain anonymous but your review may still be made public.

**Do you want your identity to be public for this peer review?**  For information about this choice, including consent withdrawal, please see our Privacy Policy

Reviewer #1: No

Reviewer #3: **Yes:**  Abduljalil Umar Abdullahi

Reviewer #4: **Yes:**  Chibuike Agu

**********

[NOTE: If reviewer comments were submitted as an attachment file, they will be attached to this email and accessible via the submission site. Please log into your account, locate the manuscript record, and check for the action link "View Attachments". If this link does not appear, there are no attachment files.]

To ensure your figures meet our technical requirements, please review our figure guidelines: https://journals.plos.org/plosone/s/figures

You may also use PLOS’s free figure tool, NAAS, to help you prepare publication quality figures: https://journals.plos.org/plosone/s/figures#loc-tools-for-figure-preparation.

NAAS will assess whether your figures meet our technical requirements by comparing each figure against our figure specifications.

# Author comment: Author response to Decision Letter 2

**DOI:** 10.1371/journal.pone.0359973.r004

19 Sep 2026

Reviewers' comments:

Please use the space provided to explain your answers to the questions above. You may also include additional comments for the author, including concerns about dual publication, research ethics, or publication ethics. (Please upload your review as an attachment if it exceeds 20,000 characters)

Reviewer #1: Please, review the writing style throughout the manuscript again to avoid repetitions and to ensure a fluent reading.

Response: Thank you for this comment. We have reviewed the manuscript again and made some changes in the introduction to limit repetitions (Line 79-102)

Reviewer #3: The research is community based and there is need to comply with National Health Research Ethics Committee of Nigeria Code of health research ethics by involving the Community before, during and after conduct of the research. If that has been done, then the researchers need to state it clearly under the section for ethical considerations.

All other comments raised during the preview have been addressed.

Response: Thank you for this important observation. Relevant community leaders and representatives were engaged before the study, supported community entry during data collection, and were informed of the findings after the study. This information has now been added to the “Ethics approval and consent to participate” section (Line 334-336).

Reviewer #4: Thank you for the opportunity to review this manuscript, which addresses an important public health issue by examining the prevalence and determinants of emotional, physical, and sexual intimate partner violence (IPV) among women in urban Lagos, Nigeria. The study is relevant to ongoing efforts to understand the burden and drivers of IPV in low- and middle-income settings and has the potential to contribute valuable evidence to inform policy and intervention strategies. The manuscript is generally well structured and the research question is of public health significance. However, several issues related to the clarity, methodological reporting, internal consistency, and presentation of results require attention before the manuscript can be considered for publication. My specific comments are provided below.

Abstract:

i. Indicate how the participants were selected (i.e., the sampling technique) in the Methods section of the abstract.

Response: Thank you for this comment. We have included the sampling technique in the abstract (Line 43).

Introduction

i. The second and third paragraphs are conceptually repetitive, as both discuss gender inequality, unequal power relations, and patriarchal norms as underlying drivers of intimate partner violence. Consider integrating these concepts into a single paragraph to eliminate redundancy, improve the flow of the introduction, and provide a smoother transition to the subsequent discussion of the burden of IPV in Nigeria.

Response: Thank you for this comment. We have modified these two paragraphs to remove repetition and redundancy (Line 79-102).

Study area

i. Consider including the literacy or educational profile of the study area, as this is an important contextual factor relevant to intimate partner violence.

ii. Please provide a brief justification for the selection of Mushin LGA as the study site by highlighting characteristics that make it relevant to the study of intimate partner violence. This will strengthen the contextual basis of the study.

Response: Thank you for this helpful comment. The Study Area section has been revised to include the educational and literacy profile of Mushin LGA. We have also clarified that Mushin has a high population density, relatively low socioeconomic status, educational challenges, diverse population and predominance of informal economic activities (Line 153-177).

Sample size

i. The rationale for inflating the sample size by 30% for non-response requires further justification. Given the interviewer-administered data collection approach, this assumption appears relatively high unless supported by evidence from previous studies, the unique logistical and ethical challenges associated with IPV research, etc.

Response to reviewer

Thank you for this observation. The 30% allowance was a conservative estimate made during the study-planning stage because the sensitive nature of IPV questions and the privacy requirements for household interviews were expected to result in refusals or incomplete interviews. However, the actual response rate was 90.6%, which was higher than anticipated. We have revised the Sample Size Determination section (Line 193-197)

Sampling methods

The multistage sampling procedure requires further clarification to ensure reproducibility. Specifically, please indicate

i. the total number of streets included in the sampling frame

ii. the number selected from each ward, together with the basis for determining this number;

iii. the procedure used to select one eligible woman within a selected household when more than one woman met the inclusion criteria;

iv. clarify the selection process in houses containing multiple households by distinguishing household selection from respondent selection.

Response: Thank you for highlighting the need for greater clarity regarding the multistage sampling procedure. Data collection was conducted in 2022, and unfortunately, the field sampling records are no longer available to verify the exact number of streets in the sampling frame, the number selected from each ward, the number of houses listed, or the basis for determining the number selected per ward. We therefore cannot provide these figures reliably and have avoided retrospectively introducing unverifiable information.

However, we have clarified that one household was selected by balloting in houses with multiple households and, where necessary, one eligible woman was similarly selected from that household(Line 205-212).

Data collection instruments and techniques

I. First, the statement that the WHO multi-country questionnaire has been "widely validated across diverse settings" is too general. Please specify the settings in which the instrument has been validated (e.g., LMICs, African countries, or settings comparable to Lagos) and provide the relevant supporting references.

II. Second, indicate the language(s) in which the interviews were conducted

III. Third, the statement that "privacy was ensured during the interviews" should be expanded to describe the specific measures taken to ensure privacy in the household setting, given the sensitive nature of IPV research.

IV. Revise the description of the pretest for clarity by stating that the questionnaire was pretested among participants in Isolo LGA, explaining the rationale for selecting the pretest site

Response: Thank you for these helpful comments. We have specified that the WHO questionnaire was developed and implemented in diverse low- and middle-income settings, including Ethiopia, Namibia and Tanzania, with the supporting reference provided. We have also stated that interviews were conducted in English or Yoruba, described the measures used to maintain privacy, and clarified that the questionnaire was pretested among eligible women in Isolo LGA because it provided also, an urban setting (Line 226-231).

Results

The Results section is not well aligned with the study objectives. Tables 3 and 4 give disproportionate attention to attitudes towards gender roles and partner communication, which are explanatory variables rather than the primary outcomes. In contrast, emotional, physical, and sexual IPV (the main outcomes of the study) are presented only as composite prevalence estimates in Table 5.

i. Since these outcomes were assessed using multiple questionnaire items, present the individual items (or a summary of them) before the composite prevalence estimates to show how the outcomes were derived.

ii. If attitudes and partner communication are not central to the study objectives, reduce their prominence in the descriptive results and focus on them in the analysis of factors associated with IPV. This will improve the coherence and alignment of the Results section with the study objectives and title.

iii. Please verify the frequencies and percentages reported for the overall attitude towards gender roles in Table 3. The reported values appear inconsistent with the study sample size (n = 502), as the frequencies do not sum to the total sample and the percentage reported for the negative attitude category does not correspond to its frequency.

Response: Thank you for this important observation. We have revised the Results section to improve its alignment with the study objectives and to give greater prominence to emotional, physical, and sexual intimate partner violence, which are the primary outcomes of the study.

i. Table 5 has been expanded to present the individual questionnaire items used to assess emotional, physical, and sexual IPV. For each item, the frequency and percentage of respondents reporting the experience during their lifetime and within the preceding 12 months are now shown. The accompanying narrative highlights the most commonly reported acts within each IPV domain. The composite prevalence estimates for emotional, physical, sexual, and any IPV are presented after the item-level findings, thereby showing more clearly how the overall IPV outcomes were derived.

ii. We agree that attitudes towards gender roles and partner communication are explanatory variables rather than the primary study outcomes. We have therefore reduced their emphasis in the accompanying narrative and clarified that they were assessed as potential determinants of IPV. The tables have been retained because both variables were included in the subsequent analysis of factors associated with IPV, and their descriptive distributions provide context for interpreting those findings. However, the revised Results section now places greater narrative emphasis on the item-level and composite IPV findings.

iii. We rechecked the analysis for overall attitude towards gender roles and identified that the frequencies previously entered in Table 3 were incorrect. The corrected results show that 7 respondents (1.4%) had a negative attitude, while 495 respondents (98.6%) had a positive attitude towards gender roles. These frequencies sum to the total sample of 502, and the corresponding percentages sum to 100.0%. Table 3 and the accompanying narrative have been corrected accordingly.

(Line 338-675)

Limitations

i. Please indicate the measures taken to mitigate recall and social desirability biases.

Response: Thank you for this comment. We have revised the Limitations section to state the measures used to minimise recall and social desirability biases, including trained female interviewers, private interviews, assurances of confidentiality, behaviourally specific questions, and clearly defined reference periods (Line 820-824).

Attachment Submitted filename: Response to Reviewers_19 09 2026.docx

# Editor report: Decision Letter 2

**DOI:** 10.1371/journal.pone.0359973.r005

**Authors:** Adewale Ashimi

21 Sep 2026

Prevalence and determinants of emotional, physical, and sexual Intimate Partner Violence in urban Lagos, Nigeria: a cross-sectional study

PONE-D-25-58389R2

Dear Dr. Olubodun,

We’re pleased to inform you that your manuscript has been judged scientifically suitable for publication and will be formally accepted for publication once it meets all outstanding technical requirements.

Within one week, you’ll receive an e-mail detailing the required amendments. When these have been addressed, you’ll receive a formal acceptance letter and your manuscript will be scheduled for publication.

An invoice will be generated when your article is formally accepted. Please note, if your institution has a publishing partnership with PLOS and your article meets the relevant criteria, all or part of your publication costs will be covered. Please make sure your user information is up-to-date by logging into Editorial Manager at Editorial Manager® and clicking the ‘Update My Information' link at the top of the page. For questions related to billing, please contact billing support.

If your institution or institutions have a press office, please notify them about your upcoming paper to help maximize its impact. If they’ll be preparing press materials, please inform our press team as soon as possible -- no later than 48 hours after receiving the formal acceptance. Your manuscript will remain under strict press embargo until 2 pm Eastern Time on the date of publication. For more information, please contact onepress@plos.org.

Kind regards,

Adewale Olufemi Ashimi, MBChB, MPH, PhD, FWACS

Academic Editor

PLOS One

Additional Editor Comments (optional):

Reviewers' comments:

# Editor report: Acceptance letter

**DOI:** 10.1371/journal.pone.0359973.r006

**Authors:** Adewale Ashimi

PONE-D-25-58389R2

PLOS One

Dear Dr. Olubodun,

I'm pleased to inform you that your manuscript has been deemed suitable for publication in PLOS One. Congratulations! Your manuscript is now being handed over to our production team.

At this stage, our production department will prepare your paper for publication. This includes ensuring the following:

* All references, tables, and figures are properly cited

* All relevant supporting information is included in the manuscript submission,

* There are no issues that prevent the paper from being properly typeset

You will receive further instructions from the production team, including instructions on how to review your proof when it is ready. Please keep in mind that we are working through a large volume of accepted articles, so please give us a few days to review your paper and let you know the next and final steps.

Lastly, if your institution or institutions have a press office, please let them know about your upcoming paper now to help maximize its impact. If they'll be preparing press materials, please inform our press team within the next 48 hours. Your manuscript will remain under strict press embargo until 2 pm Eastern Time on the date of publication. For more information, please contact onepress@plos.org.

You will receive an invoice from PLOS for your publication fee after your manuscript has reached the completed accept phase. If you receive an email requesting payment before acceptance or for any other service, this may be a phishing scheme. Learn how to identify phishing emails and protect your accounts at https://explore.plos.org/phishing.

If we can help with anything else, please email us at customercare@plos.org.

Thank you for submitting your work to PLOS One and supporting open access.

Kind regards,

PLOS One Editorial Office Staff

on behalf of

Dr. Adewale Olufemi Ashimi

Academic Editor

PLOS One
